# Transient Salt-Bridge-Based Supramolecular Polymers: Experiments and Theory

**DOI:** 10.1021/jacs.5c22087

**Published:** 2026-02-04

**Authors:** Gabriele Melchiorre, Matteo Valentini, Francesco Ranieri, Davide Cantiello, Roberta Cacciapaglia, Laura Baldini, Gianfranco Ercolani, Stefano Di Stefano

**Affiliations:** a Dipartimento di Chimica and Istituto per i Sistemi Biologici - CNR (ISB-CNR), Sede Secondaria di Roma - Meccanismi di Reazione, c/o Dipartimento di Chimica Università di Roma La Sapienza, P.le A. Moro 5, Rome I-00185, Italy; b Dipartimento di Scienze Chimiche, della Vita e della Sostenibilità Ambientale, 9370 Università degli Studi di Parma, Parco Area delle Scienze 17/A, Parma 43124, Italy; c Dipartimento di Scienze e Tecnologie Chimiche, 9318 Università di Roma Tor Vergata, Via della Ricerca Scientifica, Roma 00133, Italy

## Abstract

The smooth decarboxylation under basic conditions of activated carboxylic acids (ACAs) is exploited to achieve a transient supramolecular polymer based on hydrogen bonds reinforced by electrostatic interactions. In particular, it is proved that when the aliphatic α,ω-diamine **3**, namely, 1,8-diamino-3,6-dioxaoctane, reacts with an equimolar amount of the activated dicarboxylic acid **1**H_2_, *i.e.*, a difunctional derivative of 2-cyano-2-phenylpropanoic acid, a supramolecular polymer of the kind −AB—BA—AB– is immediately formed in chloroform solution. The A—A and B—B monomers are held together by salt bridges (hydrogen bonds reinforced by electrostatic interactions) between ammonium and carboxylate functions. The larger the concentration of the added materials, the higher the polymerization degree (DP) of the polymer. Under the given experimental protocol, such a polymer disaggregates over time due to decarboxylation, and at the end of the process, only diamine **3** and waste product **4**, which cannot interact with one another anymore, remain in the solutions. DOSY spectra recorded at different reaction times definitely demonstrate the phenomenology described above. The trend of the degree of polymerization as a function of monomer concentration has been clarified in the light of the ring–chain equilibrium theory. The application of the theory enables the accurate evaluation of the distribution of linear and cyclic oligomers as well as the critical concentration, *c*_crit_, above which polymerization rapidly becomes more extensive due to the saturation of macrocyclic species. Notably, the ACA is not used just as a stimulus for a dissipative system, but as one of its structural components.

## Graphical abstract

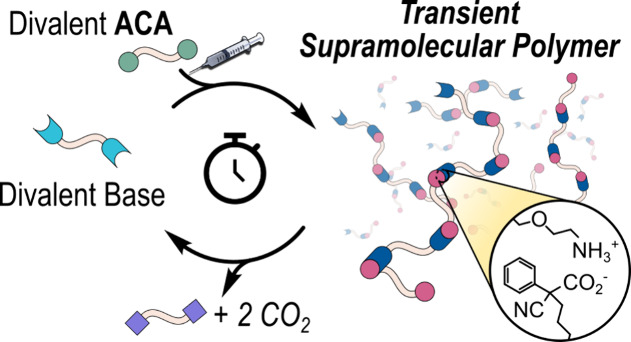

## Introduction

Chemically time-programmable systems are receiving increasing interest from the scientific community due to several reasons, including the possible achievements of (i) smart materials able to respond to external stimuli (soft robotics and self-healing polymers),^1^ (ii) artificial life-like systems able to evolve in response to changes of the environmental conditions,^2^ and (iii) molecular machines (switches and motors) capable of performing particular tasks and the like.^3^ Time programming often requires the dissipation of a chemical species, which is generally defined as a stimulus. Activated carboxylic acids (ACAs)^4^ have been recently used to program over time the operation of many chemical systems based on the acid–base reaction, ranging from host–guest pairs,^5^ catalysts,^6^ smart materials,^1f,7^ dynamic libraries,^8^ molecular machines,^3a^ ^,^ ^3c^ ^,^ ^9^ and supramolecular polymers.^10^ Such systems generally operate under dissipative conditions, with no energy transferred from the stimulus to the system;^11^ however, sometimes, this transfer occurs and an energy ratchet operates.^7c,9b,10,12^ This is the case of recently reported transient supramolecular polymers^13^ based on imine chemistry, where tribromoacetic acid (the ACA stimulus) drives a transimination reaction,^10^ which, in turn, gives rise to the formation of polymers whose monomers are held together through hydrogen bonding interactions between ammonium cations and crown-ether moieties. When the stimulus is consumed, the ammonium cations are converted to free, neutral amine functions and the polymer falls apart. However, in this case, warming of the solution is necessary to revert to the initial conditions since, in the absence of tribromoacetic acid (that is, when the stimulus is exhausted), a strong deceleration of the back-transimination necessary to restore the initial composition is observed. Tribromoacetic acid is in fact a catalyst for the back-transimination, and when it is exhausted, the system ends up in a kinetic trap.

Here, we report a new strategy for achieving a transient supramolecular polymer from two symmetrical monomers, A–A and B–B, where no stimulus is required to form or decompose the polymer. After addition, the two monomers react with each other through an acid–base reaction that activates both the immediate formation of the polymer and its slower depolymerization.

## General Design

Figure 1A shows the operation principle of a generic dissipative system driven by an ACA (RCO_2_H). Initially, a Bro̷nsted base site in the system (S) receives a proton from the ACA and passes to the protonated state SH^+^. The conjugated base of the ACA, (RCO_2_^–^), is not stable and loses CO_2_ to be transformed into a carbanion (R^–^), which is a strong base that takes back the proton from SH^+^ to restore S in the initial neutral form. In other words, the ACA is a tool to modulate over time the acidity of the environment and hence the protonation state of the basic site present in S.

**Figure 1:**
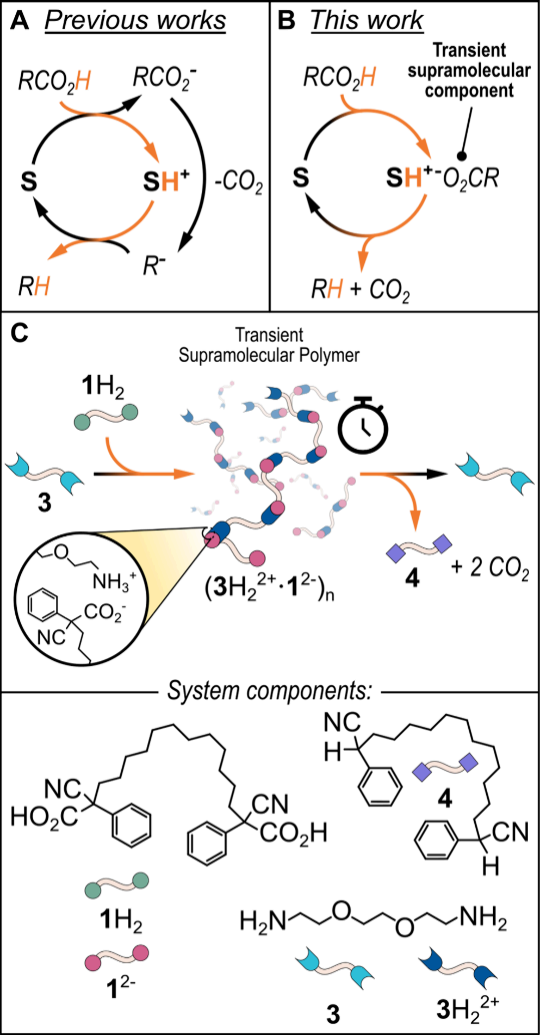
(A) Schematic representation of an acid–base-operated system driven by the decarboxylation of an ACA. (B) General operation scheme exploited in this work, in which the ACA is used as a structural component of the transient dissipative system. (C) Schematic cartoon and relative molecular structures that depict the transient formation of a salt-bridge-based supramolecular polymer generated from a difunctional ACA (**1**H_2_) and a diamine (**3**).

In the present case, the role of the ACA is different, as illustrated in Figure 1B. Here, after proton donation, its conjugate base (RCO_2_^–^) will be a structural component of the dissipative system, which will be transient in nature, given the tendency of the carboxylate anion to lose CO_2_. Thus, the ACA not only determines the temporary existence of a nonequilibrium state but also enters in the chemical structure of such non-equilibrium state as a molecular unit.^14^ More in detail, we use the difunctional ACA **1**H_2_ to transiently generate a salt-bridge-based supramolecular polymer, as shown in Figure 1C. Under basic conditions and using an aprotic solvent, **1**H_2_ should decarboxylate smoothly, exactly like its monofunctional parent 2-cyano-2-phenylpropanoic acid **2**H. The idea is to add equimolar amounts of ACA **1**H_2_ and diamine **3** in a chloroform solution to cause a proton transfer from the acidic carboxylic functions of **1**H_2_ to the basic amine functions of **3**. Such functions are now activated in the form of carboxylate anions and ammonium cations, respectively, for giving rise to a supramolecular polymer whose monomers are held together by salt bridge interactions,^15^ where hydrogen bonding is reinforced by electrostatic attraction. However, the carboxylate anions should be unstable under the adopted conditions, and due to decarboxylation and consequent back-proton transfer, the polymer should disappear over time leaving in solution neutral **3** and **4**, incapable of interacting with each other.

## Results and Discussion

### Experiments

ACA **1**H_2_ was prepared in two steps. First, commercially available ethyl 2-cyano-2-phenylacetate and 1,12-dibromododecane were reacted in DMSO (50 °C for 6 h) in the presence of K_2_CO_3_ and catalytic KI. Then, the resulting diester, purified through column chromatography (see the Supporting Information (SI) for details), was hydrolyzed in EtOH/H_2_O 15:4 (RT, for 1 day) in the presence of KOH (strong hydrogen bonding of the resulting carboxylate with the solvent protects the former from decarboxylation). Acidification with sulfuric acid causes the precipitation of **1**H_2_, which was purified and fully characterized.

A series of experiments were initially carried out in the low concentration domain to find the optimal conditions for a conveniently fast decarboxylation of **1**H_2_. The choice of the noncompetitive chloroform as a solvent was dictated by the need to favor salt-bridge interactions, on which, in our design, the supramolecular polymer is based. However, since a strong salt-bridge interaction may also retard the decarboxylation of **1**, which can become unsustainably slow, a compromise must be found.

First, 10 mM **1**H_2_ was added to 20 mM monofunctional butylamine **5** in CDCl_3_ in an NMR tube at room temperature (see Figure S18). Immediately after addition, shifts of diagnostic signals of both **1**H_2_ and **5** revealed proton transfer from the carboxylic functions of **1**H_2_ to the amino function of **5**. However, no sign of decarboxylation was detected in the following 8 h. We ascribed this result to the strong ion pairing of (**5**H^+^)_2_•**1** ^2^^–^, which retards the decarboxylation, stabilizing the carboxylate function. In fact, decarboxylation was observed to occur and finish in 2 days when carried out in the more competitive CDCl_3_/CD_3_CN 83:17 solvent mixture, where the ion pairing is weakened (see Figure S19).

To our pleasure, we discovered that when 10 mM **1**H_2_ was reacted with 10 mM diamine **3**,^16^ the decarboxylation proceeded even in pure CDCl_3_, although 5 days were needed for the complete disappearance of **1**H_2_ to give the decarboxylated product **4** (see Figure 2A,B). The higher reactivity of **1**H_2_ toward decarboxylation when **3** is used instead of **5** is very likely due to the presence of the oxygen atoms in **3**, which compete with the carboxylate functions for the hydrogen bonding with the ammonium heads.^17^

**Figure 2:**
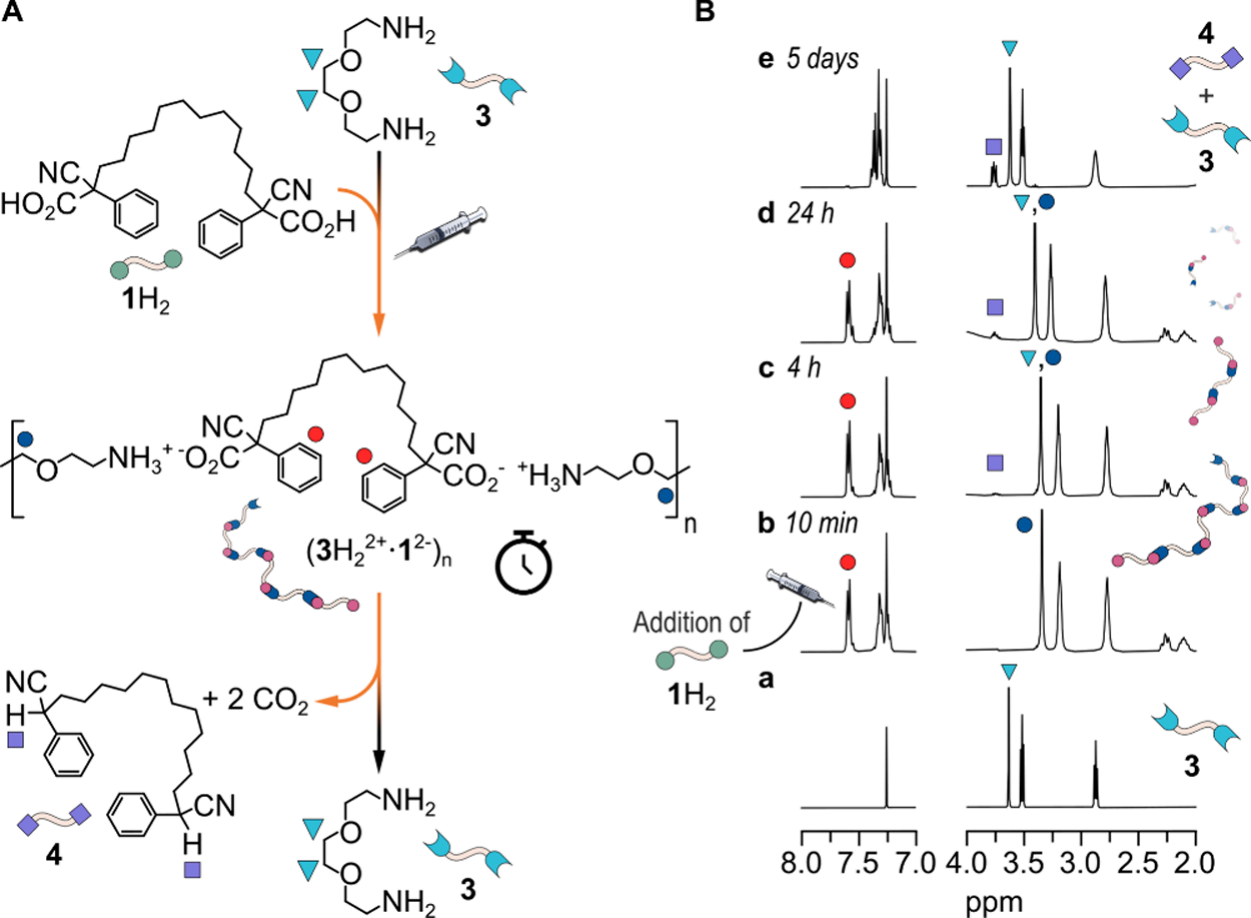
(A) Schematic representation of the diamine **3** that, after being protonated by the difunctional ACA (**1**H_2_), gives rise to a supramolecular polymer in which the bis-deprotonated ACA (**1** ^2–^) is a structural component. The decarboxylation reaction that occurs over time breaks apart the assembly, releasing the starting diamine **3** and the decarboxylated product **4**, which are unable to interact with each other. (B) ^1^H NMR monitoring of a solution containing the diamine **3** (10 mM) depicted in (A) before (trace *a*, *t* = 0) and after (from trace *b* to trace *e*) the addition of 10 mM ACA **1**H_2_ (CDCl_3_, 25 °C; see (A) for the color code), demonstrating the ability of **3** to catalyze the decarboxylation of the ACA over the time.

Trace *a* of Figure 2B is related to 10 mM **3**, while trace *b*, which is recorded 10 min after the addition of **1**H_2_, clearly shows that the acid–base reaction between **1**H_2_ and **3** has occurred (refer to Figure 2A for signal assignment). From now onward, decarboxylation occurs (traces *b* to *e*) and, after 5 days (trace *e*), the reaction is complete with the signals of **3** restored at the initial chemical shift values.

Next, a series of experiments at higher and equimolar concentrations of **1**H_2_ and **3** were performed to achieve a transient supramolecular polymer. The experimental protocol consisted of adding equimolar amounts of **1**H_2_ and **3** in CDCl_3_ in a series of NMR tubes at increasing concentration. Since the decarboxylation is slow enough (it lasts 5 days; see above), the bidimensional DOSY spectra of the different tubes taken immediately after the addition of **1**H_2_ and **3** give a measure of the initial weight-average polymerization degree (DP) of the (**3**H_2_^2+^•**1** ^2^^–^)_*n*_ polymers present in each tube.

Table 1 reports the DPs obtained from diffusion coefficients (*D*_obs_) recorded at different and equimolar concentrations following eq 1.

$$\mathrm{DP}={\left(\frac{D_{\mathrm{monomer}}}{D_{\mathrm{obs}}}\right)}^{2}$$ 1

**Table 1:** Initial Diffusion Coefficients (*D*_obs_) for 1:1 Mixtures of **1**H_2_ and **3** at Different Concentrations (*c*_0_), and Weight-Average Degree of Polymerizations (DP) Calculated by eq 1

*c*_0_ (mM)	*D*_obs_ (cm^2^/s)^a^	DP^b^
5	5.77 × 10^–6^	4.3
10	5.78 × 10^–6^	4.2
30	5.67 × 10^–6^	4.4
50	2.34 × 10^–6^	26
60	2.28 × 10^–6^	27
80	1.69 × 10^–6^	50
100	1.20 × 10^–6^	98
150	7.28 × 10^–7^	267
200	5.66 × 10^–7^	442

a Relative error is below ±2%.

b Calculated relative error is ±6%.

This last equation was derived following a recent approach,^18^ where the squared power is just an empirical value that has been shown to fit well the behavior of polymers in CDCl_3_ (see the SI for the derivation of eq 1). The squared power has also been associated with a rod-like shape of the molecules in solution, as opposed to a spherical shape that would require the cubic power.^19^ We remark, however, that the exponent in eq 1 is purely empirical, and we do not suggest any specific shape of the polymer. It is worth noting that a cubic exponent would give unreasonably high DP values.^20^ The reference *D*_monomer_ was chosen as the average of the diffusion coefficients related to 5.0 mM solutions of **1**H_2_ (*D*_obs_ = 8.10 × 10^–6^ cm^2^/s) and **3** (*D*_obs_ = 1.56 × 10^–5^ cm^2^/s), i.e., *D*_monomer_ = 1.19 × 10^–5^ cm^2^/s. A 5.0 mM solution of **4** gave a *D*_obs_ value of 8.91 × 10^–6^ cm^2^/s, very similar to that of **1**H_2_, strongly pointing to the absence of any aggregation when **1**H_2_ is the only species present in solution at a concentration as low as 5.0 mM.

After recording the DOSY spectra, the solutions were kept at room temperature for 24 h and then warmed to 50 °C to prevent the formation of a precipitate, which otherwise would be observed after 48 h, in the more concentrated samples (*c*_0_ ≥ 80 mM).^21^ The appearance of the precipitate precluded the reversibility of the system. In contrast, following the above procedure (24 h at room temperature and then heating to 50 °C), no phase separation is ever observed in any samples, and the decarboxylation is complete after a total of 48 h (thus 24 h at RT, plus 24 h at 50 °C) with high reproducibility. For example, Figure 3 reports the NMR monitoring of the reaction between 100 mM **1**H_2_ and 100 mM **3**, when the above protocol is followed (see the SI, Figures S20–S28 for the experiments at different concentrations).

**Figure 3:**
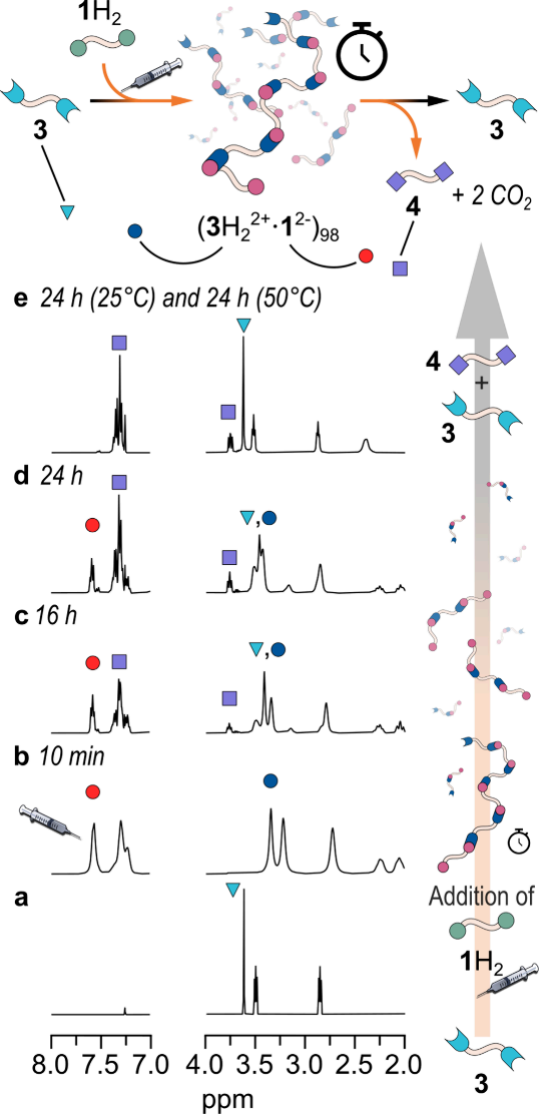
Schematic cartoon showing the transient formation of a supramolecular polymer obtained by mixing equimolar amounts of the symmetric divalent ACA **1**H_2_ and the diamine **3**. Its formation can be monitored via ^1^H NMR. Indeed, after the addition to a solution containing 100 mM diamine **3** (trace *a*, *t* = 0, blue triangle) of the ACA **1**H_2_ (from trace *b* to trace *e*), a shift in the diamine signals is observed, suggesting the formation of the supramolecular polymer ((**3**H_2_^2+^•**1** ^2^^–^)_*n*_, red and blue circles, corresponding to the difunctional ACA conjugated base and protonated diamine, respectively), which is also confirmed by DOSY experiments. Its subsequent breakdown can also be monitored by observing the appearance of the decarboxylated product **4** (violet square) over time (CDCl_3_, 25 °C for 24 h and then additional 24h at 50 °C, whole spectra in Figures S29 and S30; see the cartoon for the color code).

It is evident that at the end of the decarboxylation (after a total of 48 h), diamine **3** is recovered in its neutral form while **1**H_2_ is completely converted into product **4**. Since **3** and **4** cannot interact with each other, the supramolecular polymer disappears from the solution. Interestingly, the degree of polymerization drops to low values, long before the decarboxylation reaction is complete (vide infra). This behavior is due, not only to the decreasing concentration of active bifunctional monomers, but mainly to the intermediate formation of monofunctional monomers (semidecarboxylated and semiprotonated chains) that act as stoppers, inhibiting chain growth.

Before giving even more convincing evidence of what is occurring in the solutions, let us sum up results and explanations exposed so far: (i) a supramolecular polymer is formed when **1**H_2_ and **3** are added in equimolar amounts to the solution due to proton transfer and establishing of salt bridges; (ii) the larger the amount of equimolar **1**H_2_ + **3** added, the higher the DP of the polymer; (iii) after 24 h at room temperature and additional 24 h at 50 °C, the decarboxylation process is over and the supramolecular polymer disappears.

DOSY experiments resolved over time provide final and clear-cut evidence of our interpretation of the observed phenomenology. Figure 4a–c reports, in the typical logarithmic scale, the bidimensional DOSY spectra recorded at (a) 20 min, (b) 24 h, and (c) 48 h (with the sample held at 50 °C for the last 24 h) after mixing 200 mM **1**H_2_ and 200 mM **3** in CDCl_3_. The lowest value of *D*_obs_ is observed immediately after the addition of the materials (5.66 × 10^–7^ cm^2^/s, which corresponds to DP = 442), and then, after 24 h at room temperature, it increases to 2.20 × 10^–6^ cm^2^/s (DP = 29) and still increases to 6.72 × 10^–6^ cm^2^/s (DP = 3) after an additional 24 h at 50 °C. In other words, the supramolecular polymer (**3**H_2_^2+^•**1** ^2^^–^)_*n*_ with DP = 442, immediately formed after addition of the monomers, has completely vanished after 24 h at 25 °C and additional 24 h at 50 °C. Figure 4d and Figure 4e show the corresponding signals on a linear scale for the DOSY spectra at *t* = 20 min and *t* = 48 h, respectively (Figure 4a and Figure 4c show the same spectra on a logarithmic scale, respectively). While in the first case, a unique, slightly disperse distribution of heavy species involving both monomers is apparent, in the second case, two light noninteracting species are clearly distinguishable, corresponding to products **3** and **4**.

**Figure 4:**
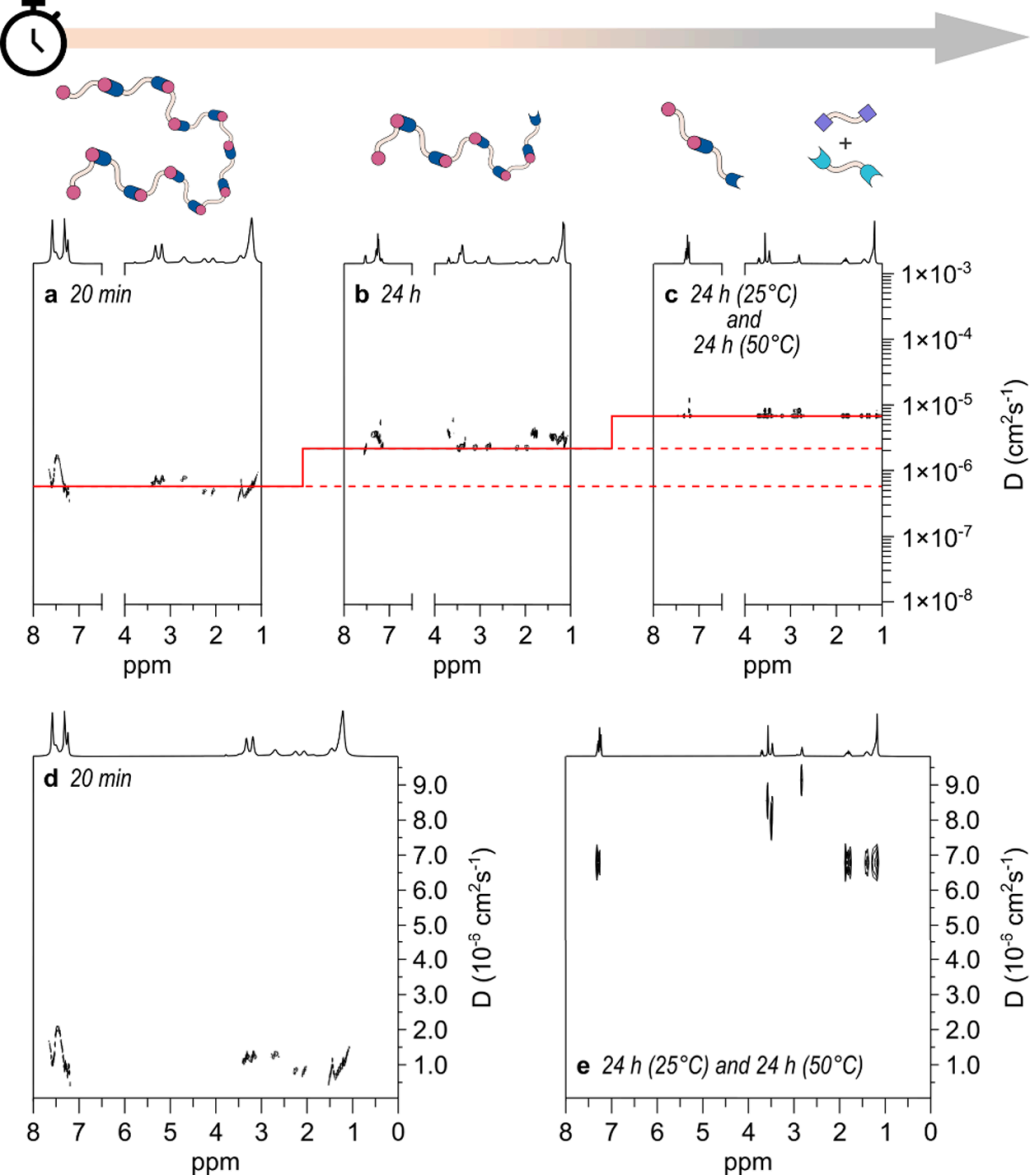
Time-resolved DOSY experiments. DOSY of a solution of 200 mM **1**H_2_ and 200 mM **3** in CDCl_3_ was taken at (a) 20 min, (b) 24 h, and (c) 48 h (with the sample held at 50 °C for the last 24 h) after mixing of the reagents on a typical logarithmic scale. Spectra (d) and (e) correspond to selected regions of spectra (a) and (c), respectively, put on a linear scale.

### Theory

The data in Table 1 show that DP increases very slowly at low monomer concentrations up to a critical concentration, around 70–80 mM, after which it increases very sharply. This behavior can be understood in the light of the theory of ring–chain equilibria presented by Jacobson and Stockmayer (JS) as early as 1950 ^22^ and successively restated to make it more understandable outside the community of polymer chemists.^23^ An even more straightforward version of the theory applied to the specific case of equimolar A—A and B—B polymerizations is reported in the SI. According to the theory, two monomers of the types A—A and B—B, capable of reacting reversibly with each other, give rise to an equilibrium mixture of linear and cyclic oligomers whose distribution depends on the monomer concentration *c*_0_, the intermolecular equilibrium constant for the reaction between the end groups *K*, and the effective molarities EM_*i*_ of the rings being formed. The latter parameter is defined as the ratio *K*_(intra)*i*_/*K*, where *K*_(intra)*i*_ is the intramolecular equilibrium constant for the ring-closing reaction leading to the cyclic *i-*mer. It measures the cyclization tendency of the linear precursor of the ring independently of the reactivity of its end groups.^24^ JS have shown that for a series of strainless cyclic oligomers formed from long chains obeying Gaussian statistics (say longer than 25–30 skeletal bonds), the equilibrium effective molarity, EM_*i*_ varies inversely with the 5/2 power of the oligomerization degree as shown by eq 2, where the factor *B* corresponds to the effective molarity of the smallest cyclic oligomer.^22,23^

$${\mathrm{EM}}_{i}=Bi^{-5/2}$$ 2

In the present case, all of the cyclic oligomers *c*-(**1** ^2–^·**3**H_2_^2+^)_*i*_ are large enough to follow eq 2. Accordingly, the mass balance equation in terms of the monomeric units of one kind is given by eq 3, where *x* is the extent of reaction in the chain fraction.

$$c_{0}=B\overset{\infty }{\underset{i=1}{\sum }}i^{-3/2}x^{2i}+\frac{x}{2K{\left(1-x\right)}^{2}}$$ 3

The two terms in the right-hand side of eq 3 represent the amount of monomer of one kind in the ring (*c*_r_) and chain (*c*_c_) fractions, respectively. When the intermolecular constant *K* is very large, eq 3 predicts the phenomenon of the critical concentration, *c*_crit_. It is the monomer concentration below which all the monomers go into the cyclic fraction, and above which the cyclic fraction stops growing and all the monomers go into the chain fraction.^22,23^ This phenomenon occurs because a very large *K* value assures that *c*_c_ is always negligible until *x* is very close to 1, whereas *c*_r_ converges to *c*_crit_ = 2.612*B* for *x* tending to 1,^25^ meaning that the cyclic fraction, in contrast with the chain fraction, can only contain a limited number of monomeric units.

JS have shown that for equimolar concentrations of A—A and B—B (the case they call the “equivalent” polymer), the weight-average degree of polymerization is given by eq 4, where DP_r_ and DP_c_, given by eqs 5 and 6, are the weight-average degrees of polymerization of the ring and chain fractions, respectively (see also the SI for the derivation of eqs 4–6).

$$\mathrm{DP}=\frac{c_{r}{\mathrm{DP}}_{r}+c_{c}{\mathrm{DP}}_{c}}{c_{0}}$$ 4

$${\mathrm{DP}}_{r}=\frac{2\sum_{i=1}^{\infty }i^{-1/2}x^{2i}}{\sum_{i=1}^{\infty }i^{-3/2}x^{2i}}$$ 5

$${\mathrm{DP}}_{c}=\frac{1+x}{1-x}$$ 6

Considering eqs 3 and 4, the data of DP vs *c*_0_ from Table 1 have been fitted by a least-squares procedure to optimize the values of *B* and *K* [optimized values are *B* = (3.1 ± 0.1) × 10^–2^ mol L^–1^ and *K* = (4.4 ± 0.1)×10^5^ mol^–1^ L; see the SI for details]. The fit of the calculated curve to the experimental data, shown in Figure 5, is remarkably good, supporting eq 4 and the presence of ring–chain equilibria in solution. The calculated critical concentration, *c*_crit_ = 8.1 × 10^–2^ mol L^–1^, corresponds to the *c*_0_ value where the chain fraction steeply grows.

**Figure 5:**
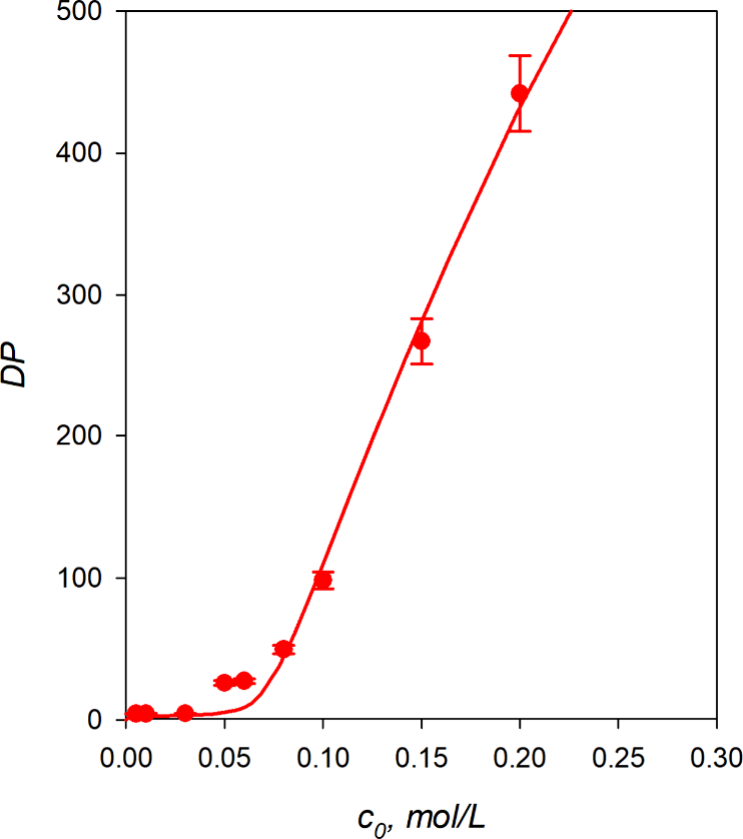
Plot of the initial weight-average degree of polymerization, DP, against the monomer concentration, *c*_0_. The points are experimental, and the curve is calculated using eqs 3 and 4 with the optimized values of *B* and *K.*

It is worth noting that in the case of ring–chain equilibria, the number-average degree of polymerization, in contrast to the weight-average degree of polymerization, is always very low. Indeed, at high *c*_0_ values, the ring fraction is scarcely significant on a weight basis but makes a substantial contribution to the total number of molecules and, hence, markedly lowers the overall number-average degree of polymerization. This will be particularly true when *x* is close to 1 and the number of chain polymer molecules is consequently small.

One might wonder how significant the values of *B* and *K* obtained from the fit are. Regarding the value of *B*, it is interesting to compare the obtained value with that estimated by a practical method suggested by Mandolini for large rings (see the SI for details).^24c^ ^,^ ^24d^ ^,^ ^24f^ The method is based on eq 7, where ν is the number of rotatable single bonds present in the smallest cyclooligomers and σ is its symmetry number accounting for the number of equivalent bonds available for ring opening.

$$B=\frac{6.63}{\sigma }\nu^{-3/2}$$ 7

In the present case, ν = 24 and σ = 2; thus, a value of *B* = 2.8 × 10^–2^ mol L^–1^ can be calculated, which is in very good agreement with that obtained by fitting the experimental DP values. As to the value of *K*, we tried to measure its value directly by titrating butylamine **5** with **2**H in deuterated chloroform at room temperature. The association constant between **5**H^+^ and **2** ^–^ is so strong that only a lower limit for *K* (≥10^5^ mol^–1^ L) could be estimated (see Figure S34). The same result was found when diamine **3** was rapidly (to avoid decarboxylation) titrated with monofunctional acid **2**H. Again, the binding (1:2) between **3**H_2_^2+^ and **2** ^–^ was too strong to measure (see Figure S36 and related details). However, this limit is in full accordance with the value of *K* obtained by the fitting procedure.

## Conclusions

In this report, we show that a transient, salt-bridge based supramolecular polymer can be obtained by the simple reaction of equimolar amounts of a diamine and a divalent activated dicarboxylic acid. Upon addition of the reagents, the supramolecular polymer is initially formed and then slowly decomposes due to decarboxylation under the given experimental conditions. Time-resolved DOSY spectroscopy allows the polymer’s disaggregation to be followed over time. The experimental data recorded immediately after the addition of the reagents allowed for the determination of the initial polymerization degree as a function of the monomer concentration. The obtained curve is fitted remarkably well by a model of reversible polymerization that accounts for the formation of cyclic oligomers. To the best of our knowledge, such a fitting has no precedent in the literature. The model, initially proposed by Jacobson and Stockmayer,^22^ and subsequently revisited to highlight the role played by the equilibrium constant *K* for linear propagation,^23^ is presented here for the specific case of equimolar A—A and B—B polymerizations, in a version that should be easily understandable even by nonexperts.

It is remarkable that an activated dicarboxylic acid has been used here, not only as a stimulus to control a dissipative polymer over time but also as a building block of the polymer itself. Finally, given the importance of salt-bridge interactions in biochemistry, particularly in the stabilization of protein structures,^26^ the possible use of ACAs to temporarily control their conformations should not be overlooked.

## Supplementary Material



## Notes

Instruments, materials and methods, synthesis, NMR, UV–Vis, and FTIR-ATR spectra of newly synthesized compounds, NMR kinetic spectra, DOSY spectra, NMR titrations, theory of ring–chain equilibria, estimation of *B* and *K* values, and derivation of eq 1 (PDF)

## Abbreviations

ACA: activated carboxylic acid
^1^H NMR: proton nuclear magnetic resonance
DP: polymerization degree
2D-DOSY: two-dimensional diffusion-ordered spectroscopy
