# Supplementary material for: Transient Salt-Bridge-Based Supramolecular Polymers: Experiments and Theory
Source: J Am Chem Soc. 2026 Feb 4;148(6):6638–45. doi: 10.1021/jacs.5c22087 (PMC12921869; doi:10.1021/jacs.5c22087)
Supplement: Supplementary file 1 [file ja5c22087_si_001.pdf]

Supporting Information for

Transient Salt-Bridge Based Supramolecular Polymers: Experiments and Theory

Gabriele Melchiorre,[a] Matteo Valentini,[a] Francesco Ranieri,[a] Davide Cantiello,[a] Roberta Cacciapaglia,[a] Laura Baldini,[b] Gianfranco Ercolani,[c] and Stefano Di Stefano*[a]*

[a]Dipartimento di Chimica and Istituto per i Sistemi Biologici - CNR (ISB-CNR), Sede Secondaria di Roma - Meccanismi di Reazione, c/o Dipartimento di Chimica Università di Roma La Sapienza, P.le A. Moro 5, I-00185 Rome, Italy. E-mail: stefano.distefano@uniroma1.it

[b]Dipartimento di Scienze Chimiche, della Vita e della Sostenibilità Ambientale, Università degli Studi di Parma, Parco Area delle Scienze 17/A, 43124 Parma, Italy.

[c]Dipartimento di Scienze e Tecnologie Chimiche, Università di Roma Tor Vergata, Via della Ricerca Scientifica, 00133 Roma, Italy, E-mail: ercolani@uniroma2.it

Experimental section S4
Instruments, methods and materials S4
Synthesis and characterization of **1**H_2 ....S4
^{1}H-NMR spectrum of **1**H_2 ....S6
^{13}C-NMR spectrum of **1**H_2 ....S6
HSQC spectrum of **1**H_2 ....S7
HR-MS (ESI-TOF) analysis of **1**H_2 ....S8
UV-Vis absorption spectrum of **1**H_2 ....S9
FTIR-ATR spectrum of **1**H_2 ....S9
DOSY spectrum of 200 mM **1**H_2 ....S10
DOSY spectrum of 100 mM **1**H_2 ....S10
DOSY spectrum of 5 mM **1**H_2 ....S11
Synthesis and characterization of **4** S11
^{1}H-NMR spectrum of **4** S12
^{13}C-NMR spectrum of **4** S13
HSQC spectrum of **4** S13
HR-MS (ESI-TOF) analysis of **4** S14
DOSY spectrum of 5 mM **4** S15
DOSY spectrum of 5 mM **3** S16
^{1}H-NMR monitoring of a 1:2 mixture of **1**H_2 (10 mM) and **5** (20 mM) S17
^{1}H-NMR monitoring of a 1:1 mixture of **1**H_2 (10 mM) and **3** (10 mM) S18
DOSY spectrum of **1**H_2 (200 mM) + **3** (200 mM) S18
DOSY spectrum of **1**H_2 (150 mM) + **3** (150 mM) S19
DOSY spectrum of **1**H_2 (100 mM) + **3** (100 mM) S19
DOSY spectrum of **1**H_2 (80 mM) + **3** (80 mM) S20

DOSY spectrum of **1**H_2 (60 mM) + **3** (60 mM) ……………………….………………………………S20

DOSY spectrum of **1**H_2 (50 mM) + **3** (50 mM) ……………………………………………………….S21

DOSY spectrum of **1**H_2 (30 mM) + **3** (30 mM) ……………………………………………………….S21

DOSY spectrum of **1**H_2 (10 mM) + **3** (10 mM) ……...……………………..………………………….S22

DOSY spectrum of **1**H_2 (5 mM) + **3** (5 mM) …….……….………………..………………………….S22

^{1}H-NMR monitoring of a 1:1 mixture of **1**H_2 (100 mM) and **3** (100 mM) ……………...………….S23

^{1}H-NMR monitoring of a 1:1 mixture of **1**H_2 (100 mM) and **3** (100 mM) at 50 °C ……...…………S24

DOSY monitoring of a 1:1 mixture of **1**H_2 (100 mM) and **3** (100 mM) at 50 °C ……………...……S24

^{1}H-NMR monitoring of a 1:1 mixture of **1**H_2 (200 mM) and **3** (200 mM) at 50 °C ……...…………S25

^{1}H-NMR titration of **5** with **2**H ($CDCl_3$, RT) ……...……………………………………...………S26

^{1}H-NMR titration of **3** with **2**H ($CDCl_3$, RT) ……...……………………………………...………S28

Theory of ring-chain equilibria for an equimolar mixture of A-A + B-B monomers ……..………..S30

Estimation of the B factor by Mandolini's method ……………….………………………………..S36

Optimization of the B factor and the intermolecular equilibrium constant *K* …………..…………S37

Derivation of eq 1 ……………….……………………………………………..………………….……S42

Experimental section

Instruments, methods and materials

Monodimensional ^{1}H-NMR, ^{13}C-NMR and DOSY spectra were recorded at room temperature on a Bruker Avance III 400 MHz spectrometer. The spectra were internally referenced to the residual proton signal of the solvent at 7.26 ppm in $CDCl_3$. In $CDCl_3/CD_3CN$ 83:17 solvent, the spectra were internally referenced to the residual proton signal of the CD_3CN, set at 2.10 ppm. ^{13}C-NMR spectra were internally referenced to the ^{13}C-signal of $CDCl_3$, set at 77.16 ppm in $CDCl_3$. DOSY spectra were processed by using both MestReNova x64 and Bruker TopSpin 4.4.0. $CDCl_3$ was preliminarily filtered over a short pad of basic alumina to remove acidic impurity. Then, it was treated one night with activated molecular sieves (3 Å) to reduce the water content. However, no significant difference on the phenomenology (reaction kinetics and DP measurements) was observed when the same batch of chloroform was used without treatment with molecular sieves.

High-resolution mass spectrometry analyses were performed using a ZenoTOF 7600 instrument equipped with an ESI source. Samples were introduced into the mass spectrometer ion source by injection from the autosampler. Infrared spectra were acquired in attenuated total reflection (ATR) using a Nicolet 6700 (Thermo Fisher Scientific, Waltham, MA, USA) equipped with a Golden Gate single reflection diamond ATR accessory.

All reagents and solvents were purchased from Sigma Aldrich or TCI-chemicals.

Synthesis and characterization of $1H_2$

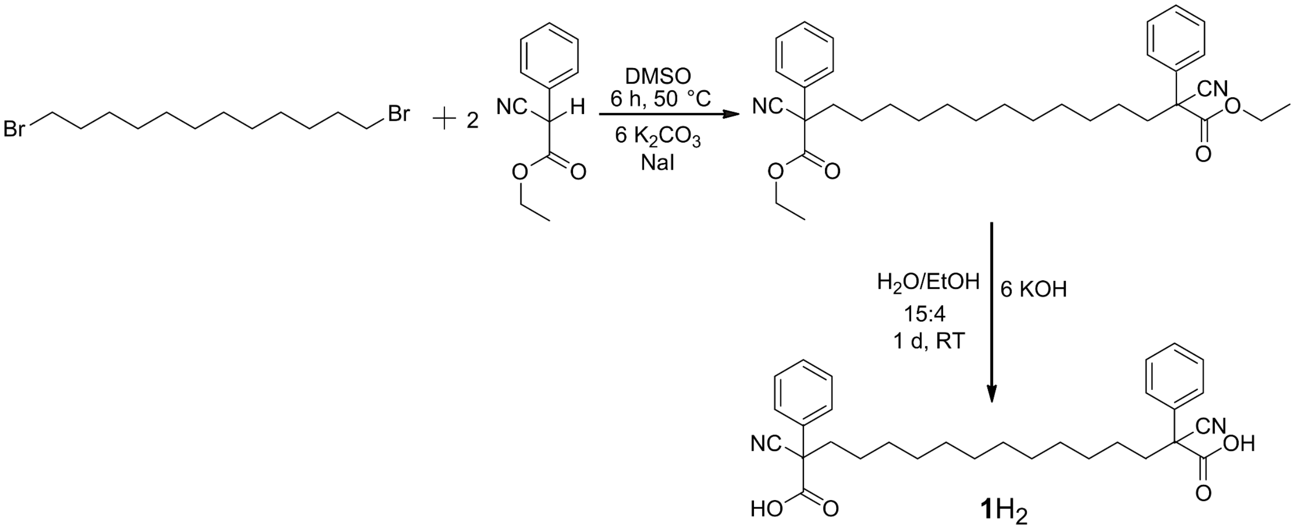

Figure S1. Synthesis of divalent ACA $\mathbf{1}H_2$.

In a three-neck round-bottom flask, approximately 7.2 mL of anhydrous DMSO were added under anhydrous conditions. Ethyl cyano(phenyl)acetate (1 mL, 5.76 mmol) was added to the solvent under an argon atmosphere and magnetic stirring, and then 1,12-dibromododecane (945 mg, 2.88 mmol), K_2CO_3 (2.39 g, 17.29 mmol), and a spatula tip of NaI were added in sequence. The reaction mixture was then heated to 50 °C under magnetic stirring for 6 hours. After that, the solution was cooled down to RT and diluted with 30 mL of water. The aqueous solution was extracted three times with 30 mL of diethyl ether. The organic fractions were mixed together and washed three times with 90 mL of a brine solution. The organic solution was dried over anhydrous Na_2SO_4 for 10 minutes, filtered, and then dried under vacuum.

The obtained crude was purified using column chromatography (SiO_2; ethyl acetate-hexane-1:5). The obtained diester (780 mg, 1.42 mmol, 50% yield) were dissolved in 3.6 mL of a H_2O-EtOH 15:4 solution with KOH (8.52 mmol, 6 equivalents). The solution was magnetically stirred overnight at RT. After that, ethanol was removed under vacuum from the solution, which was subsequently diluted with 14 mL of brine and extracted three times with 18 mL of diethyl ether. A 4 M H_2SO_4 solution was added dropwise to the aqueous phase at 0 °C, obtaining a white precipitate. The mixture was extracted three times with 70 mL of diethyl ether. The collected organic phases were dried over anhydrous Na_2SO_4, filtered, and dried under vacuum, yielding divalent ACA **1**H_2, (96% pure), as a colorless, glue-like solid. To remove occluded traces of solvents (ethanol and diethyl ether), the solid was dried under high vacuum at 50 °C for 3 days. The compound characterized by ^{1}H-NMR (Figure S2), ^{13}C-NMR (Figure S3), HSQC (Figure S4), HR-MS (Figure S5), UV-Vis absorption spectrophotometry (Figure S6), and FTIR-ATR (Figure S7). **^{1}H-NMR** (400 MHz, $CDCl_3$): δ 10.01 (s, 2H), 7.65 – 7.50 (m, 4H), 7.41 (m, 6H), 2.38 (m, 2H), 2.12 (m, 2H), 1.56 – 1.15 (m, 20H). **^{13}C-NMR** (100 MHz, $CDCl_3$): δ 173.24, 173.22, 133.84, 133.83, 129.4, 129.3, 126.37, 126.36, 117.97, 117.96, 54.5, 37.69, 37.65, 29.48, 29.46, 29.28, 29.25, 29.18, 29.15, 29.12, 29.06, 25.5, 25.4. **HR-MS (ESI-TOF)**: *m/z* calcd for $C_{28}H_{36}N_2Na$ $[M{-}2CO_2{+}Na]^+$ 423.2776; found: 423.2774. **UV-Vis**: λ [nm] (ε [$M^{-1}cm^{-1}$]) 251 (680); 258 (700); 264 (630); 280 (340). **FTIR-ATR**: $\bar{\nu}$ [cm^{-1}], 3070, 2928, 2855, 2253, 1751, 1715, 1450, 1233, 1203, 906, 766, 721, 694.

^{1}H-NMR spectrum of 1H$_2$

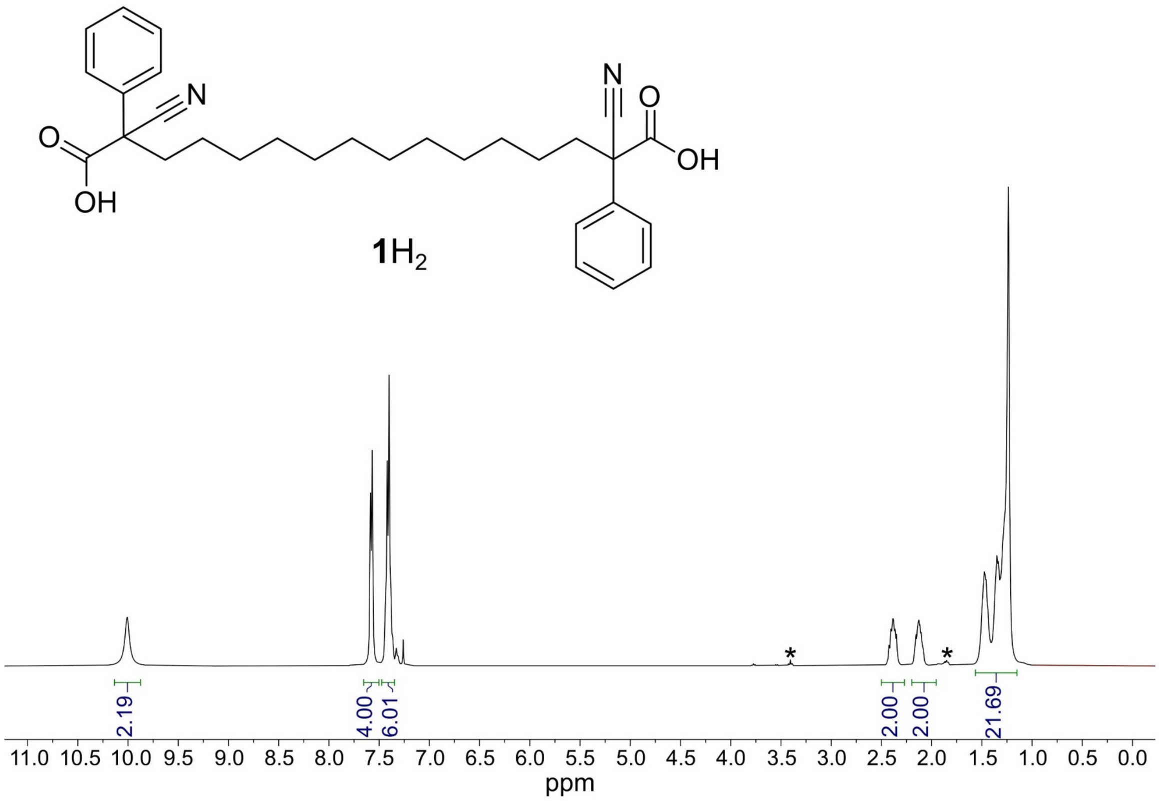

Figure S2. ^{1}H-NMR spectrum (400 MHz) of **1**H$_2$, in CDCl$_3$ (25 °C). Signals marked with an asterisk belong to an impurity, identified as the monocarboxylic acid monobromo derivative of 1,12-dibromododecane.

^{13}C-NMR spectrum of 1H$_2$

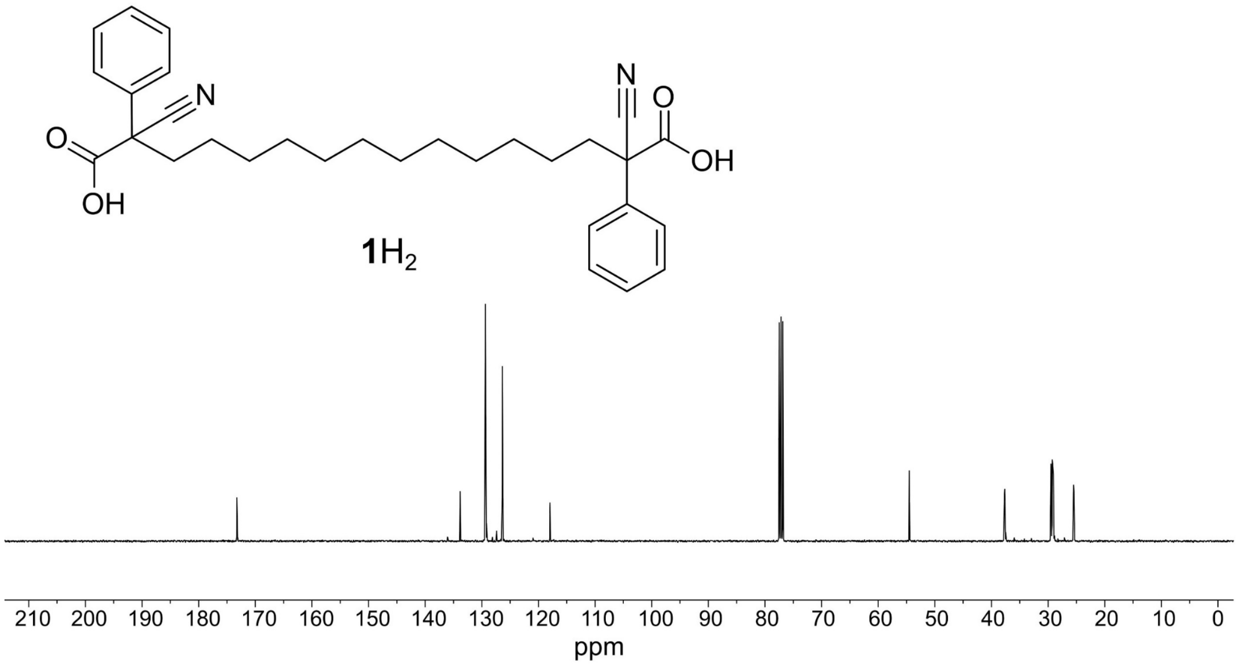

Figure S3. ^{13}C-NMR spectrum (100 MHz) of **1**H$_2$, in CDCl$_3$ (25 °C).

HSQC spectrum of 1H2

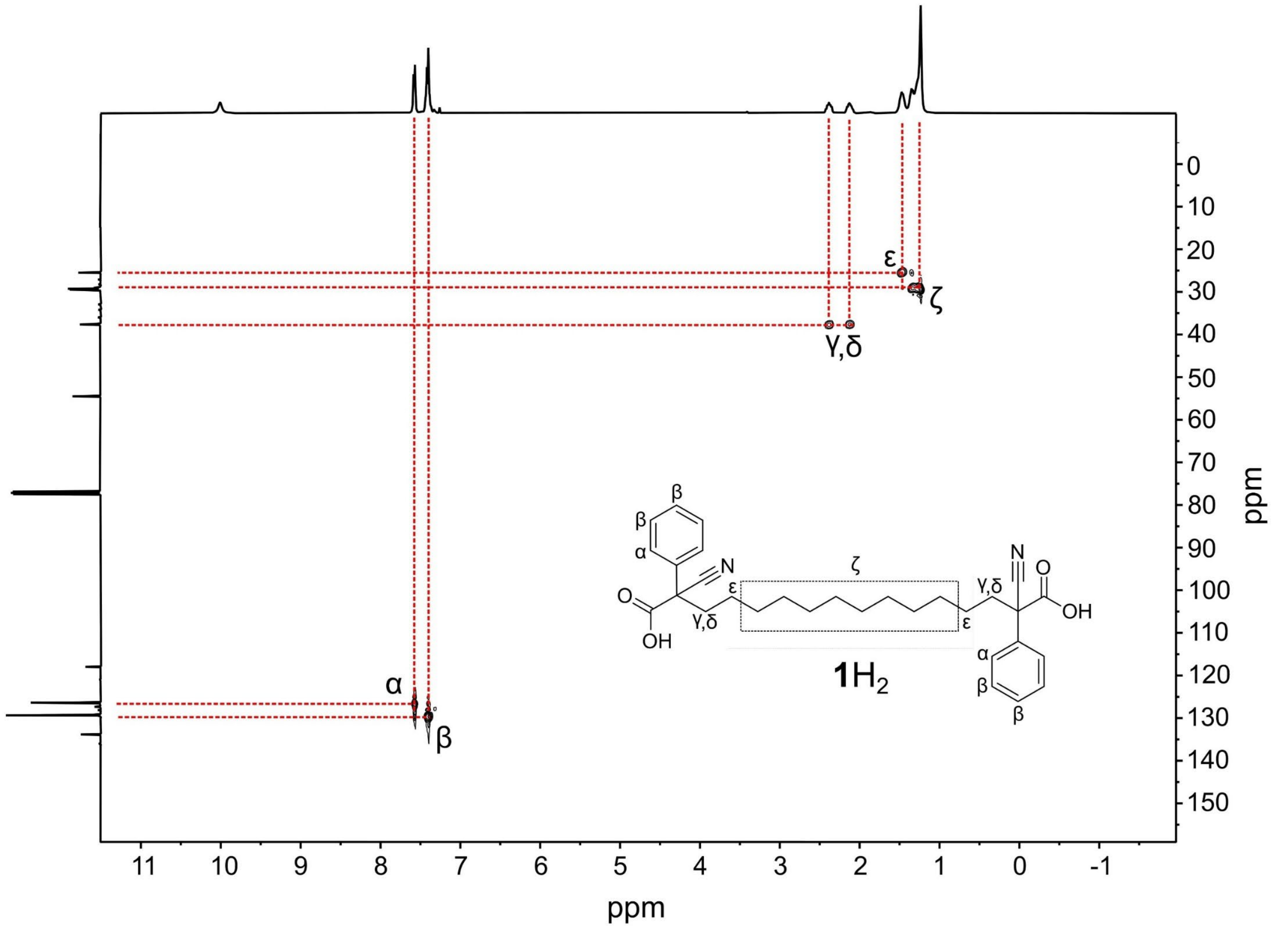

Figure S4. HSQC spectrum of **1**H_2**,** in $CDCl_3$ (25 °C).

HR-MS (ESI-TOF) analysis of $1H_2$

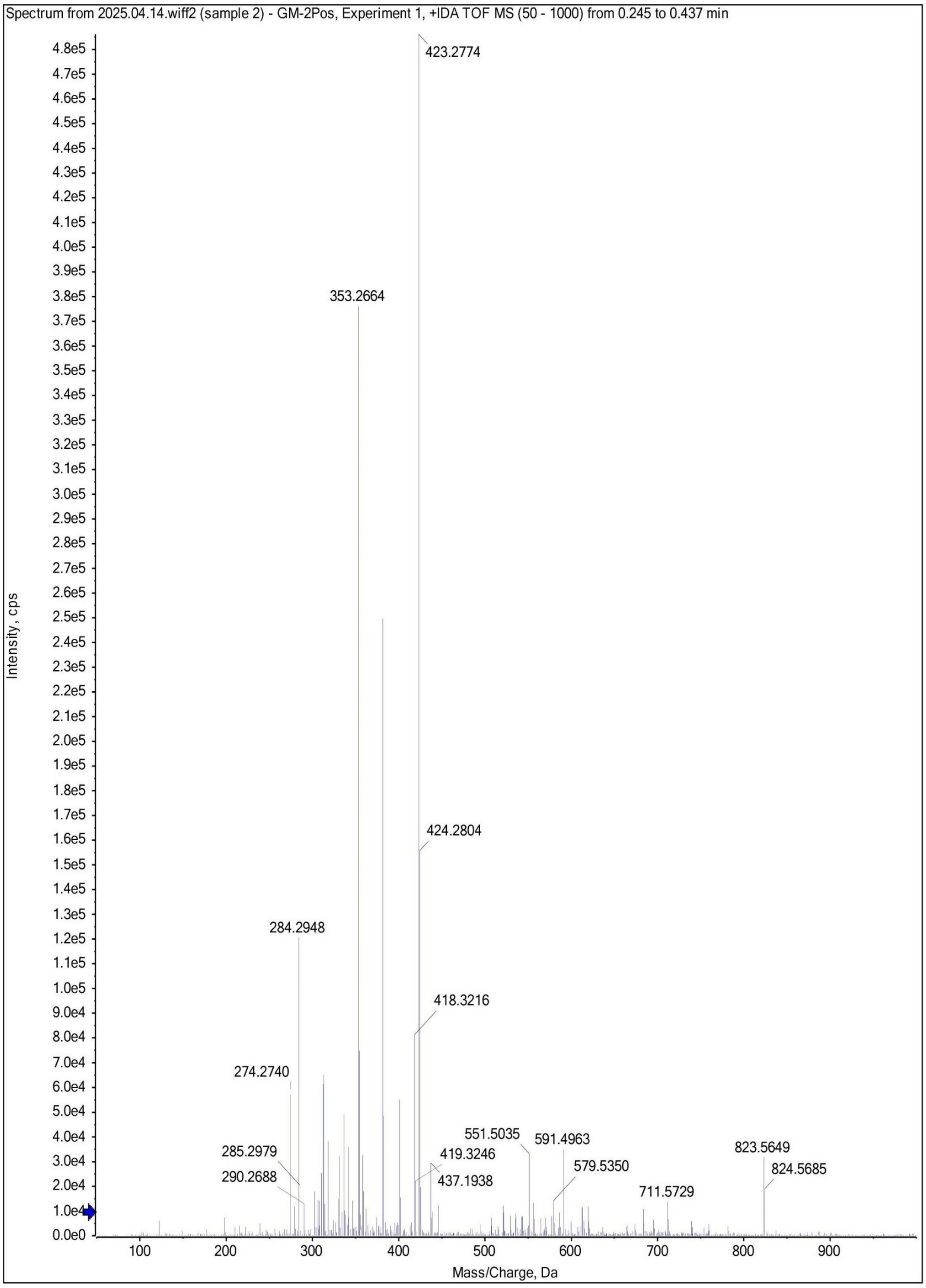

Figure S5. HR-MS (ESI-TOF) analysis of **1**H_2, (positive mode).

UV-Vis absorption spectrum of 1H2

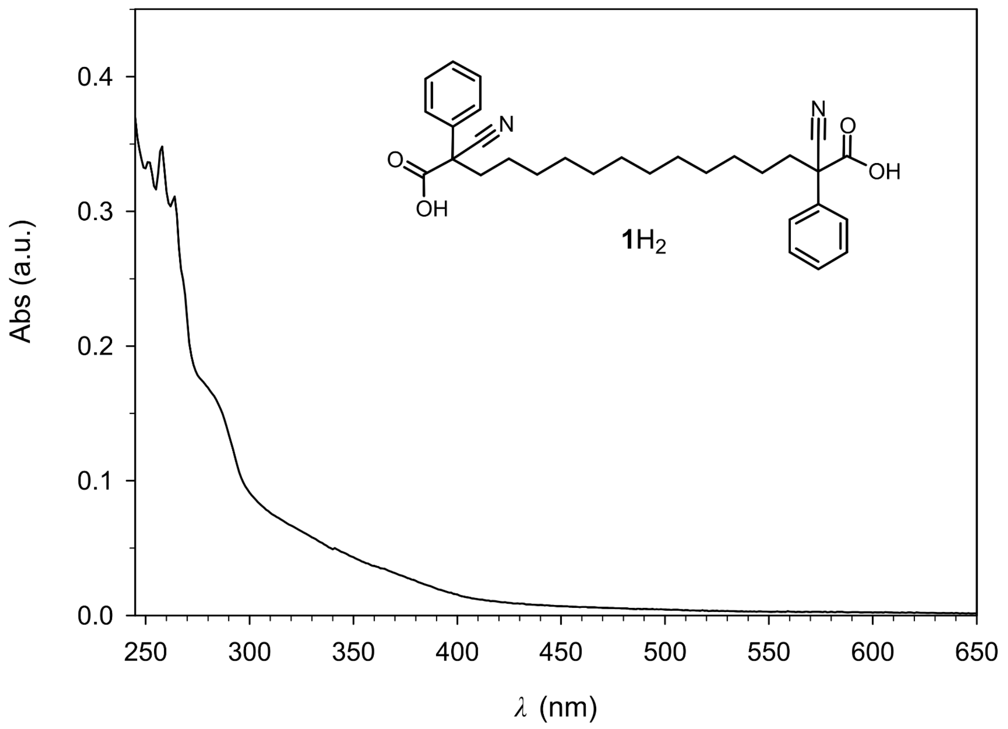

Figure S6. UV-Vis spectrum of 0.5 mM **1**H_2**,** in $CDCl_3$ (25 °C).

FTIR-ATR spectrum of 1H2

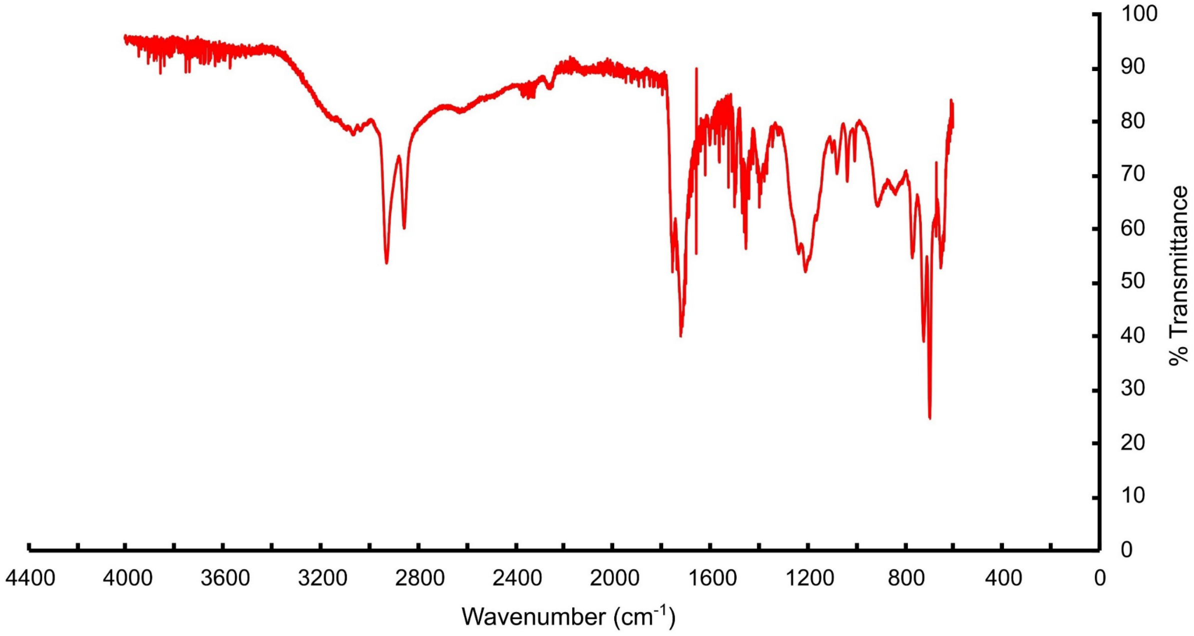

Figure S7. FTIR-ATR spectrum of **1**H_2, at 25 °C.

DOSY spectrum of 200 mM 1H2 in CDCl3

In a 1.5 mL vial, **1**H_2 (53.7 mg, 0.11 mmol) was dissolved in 550 μL of $CDCl_3$. Then, a ^{1}H-DOSY of the solution was recorded. The observed diffusion coefficient was $2.94 \cdot 10^{-6}$ cm^2/s.

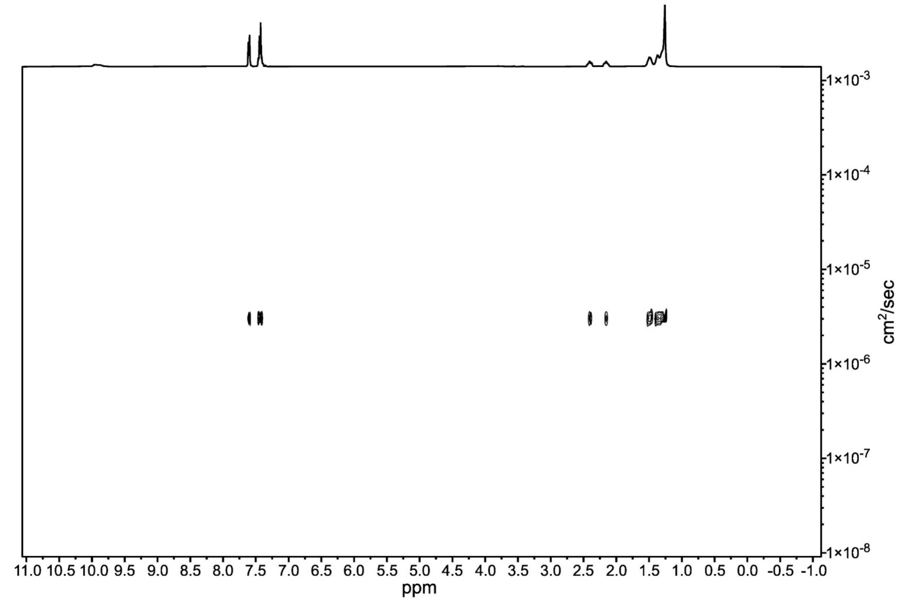

Figure S8. ^{1}H-DOSY spectrum of 200 mM **1**H_2, in $CDCl_3$ (25 °C).

DOSY spectrum of 100 mM 1H2

In a 1.5 mL vial, **1**H_2 (26.9 mg, 0.055 mmol) was dissolved in 550 μL of $CDCl_3$. Then, a ^{1}H-DOSY of the solution was recorded. The observed diffusion coefficient was $4.55 \cdot 10^{-6}$ cm^2/s.

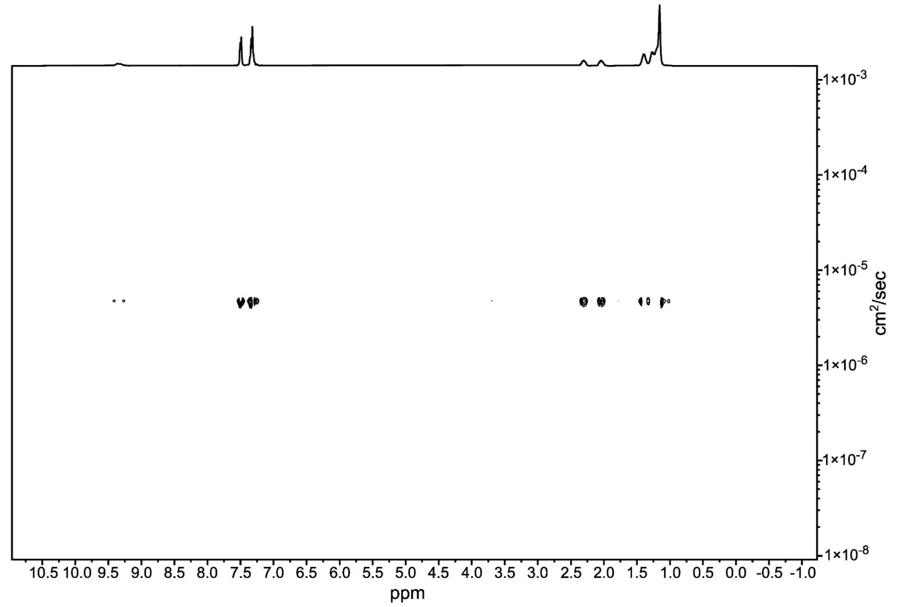

Figure S9. ^{1}H-DOSY spectrum of 100 mM **1**H_2, in $CDCl_3$ (25 °C).

DOSY spectrum of 5 mM 1H2

In a 1.5 mL vial, **1**H_2 (1.35 mg, 0.00275 mmol) was dissolved in 550 μL of $CDCl_3$. Then, a ^{1}H-DOSY of the solution was recorded. The observed diffusion coefficient was $8.10 \cdot 10^{-6}$ cm^2/s.

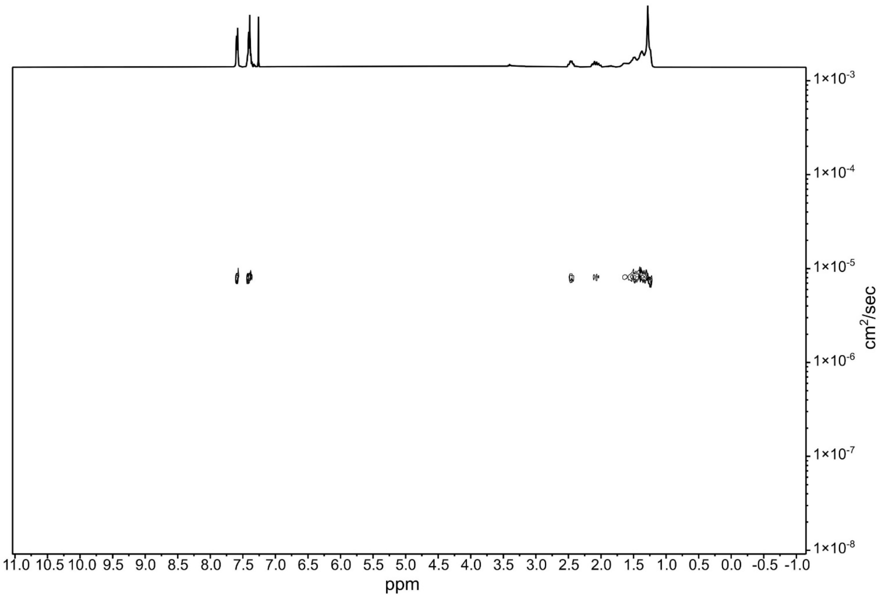

Figure S10. ^{1}H-DOSY spectrum of 5 mM **1**H_2, in $CDCl_3$ (25 °C).

Synthesis and characterization of 4

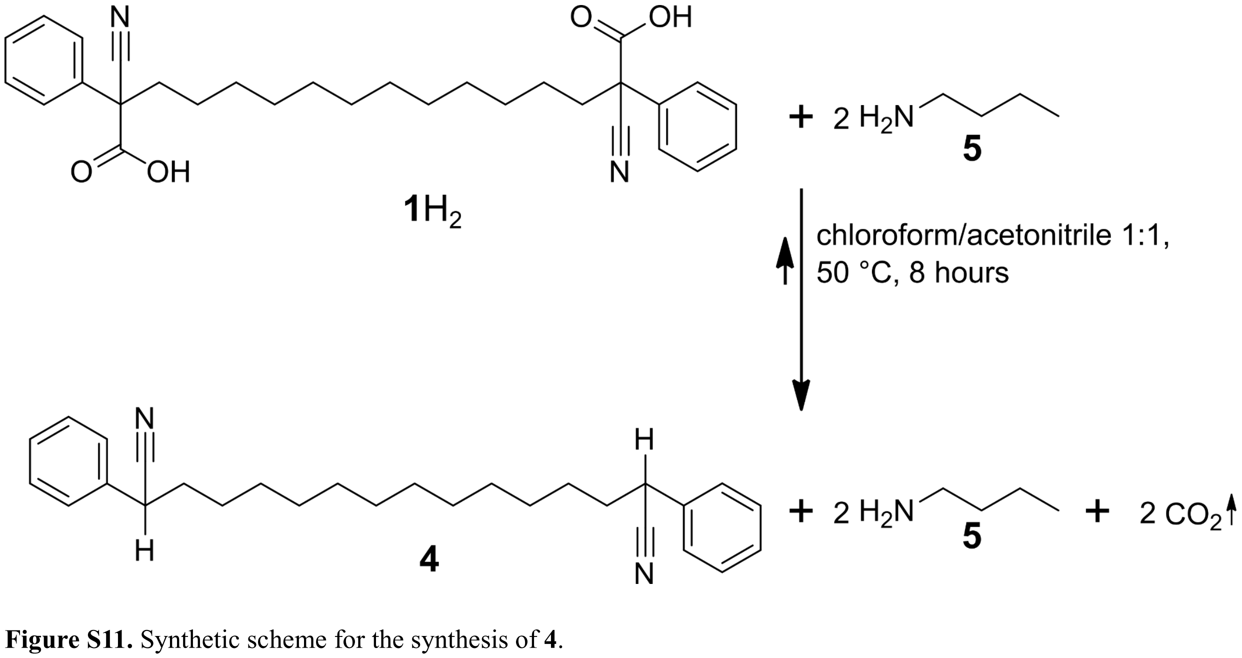

Figure S11. Synthetic scheme for the synthesis of **4**.

In a 1.5 mL vial, 300 µL of a 60 mM solution of **1**H_2 in chloroform/acetonitrile (1:1) were mixed with 300 µL of a 180 mM solution of **5** in the same solvent mixture. The resulting solution of 30 mM **1**H_2 and 90 mM **5** (50% excess) was heated at 50 °C for 4 hours, and then the solvent and **5** were removed under vacuum. The resulting product was dissolved in 600 µL of $CDCl_3$ and ^{1}H-NMR, ^{13}C-NMR and HSQC spectra were recorded. An HR-MS analysis was performed. **^{1}H-NMR** (400 MHz, $CDCl_3$): δ 7.42 – 7.27 (m, 10H), 3.78 (d, J = 6.1 Hz, 1H), 3.75 (d, J = 6.1 Hz, 1H), 2.00 – 1.80 (m, 4H), 1.56 – 1.37 (m, 4H), 1.4 – 1.2 (m, 16H). **^{13}C-NMR** (100 MHz, $CDCl_3$): δ 136.2, 129.2, 128.1, 127.4, 121.1, 37.6, 36.1, 29.63, 29.58, 29.4, 29.1, 27.2. **HR-MS (ESI-TOF)**: *m/z* calcd for $C_{28}H_{36}N_2Na$ $[M + Na]^+$: 423.2776; found: 423.2771.

^{1}H-NMR spectrum of 4

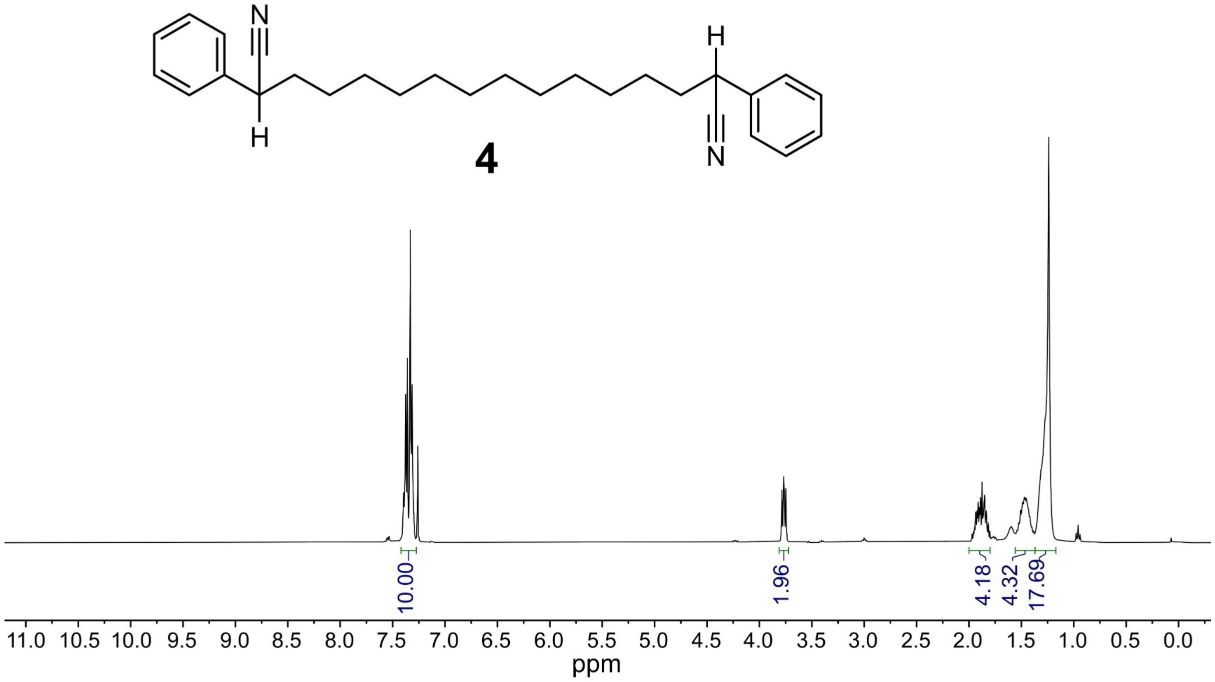

Figure S12. ^{1}H-NMR spectrum of **4** in $CDCl_3$ (25 °C).

^{13}C-NMR spectrum of 4

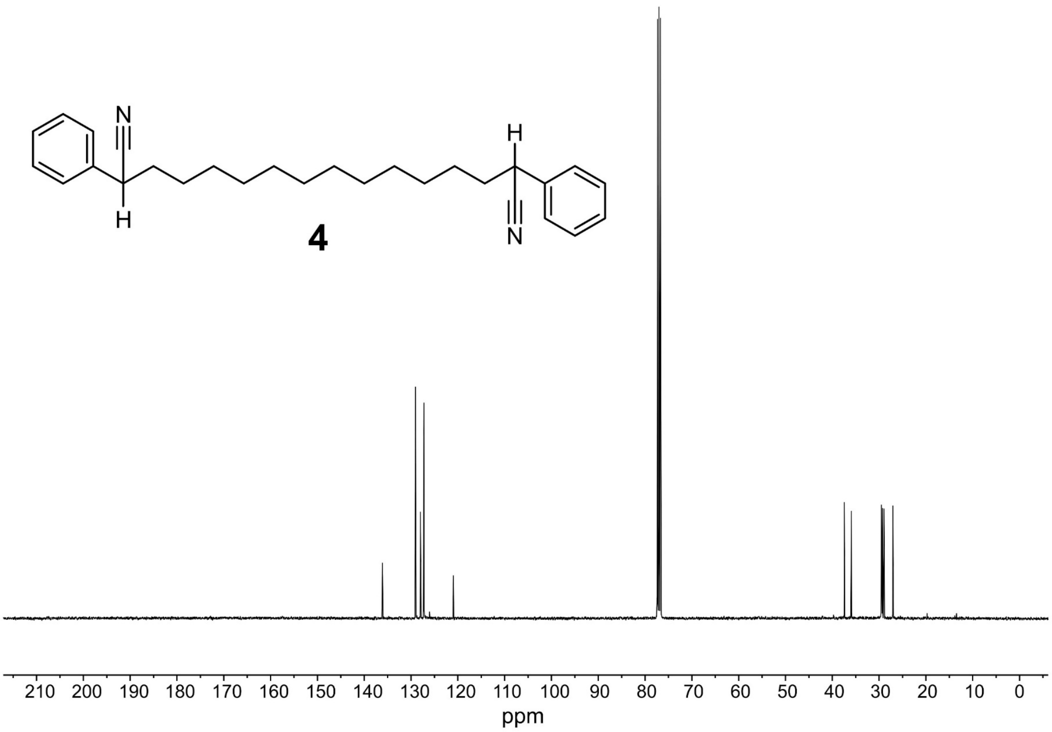

Figure S13. ^{13}C-NMR spectrum of **4** in $CDCl_3$ (25 °C).

HSQC spectrum of 4

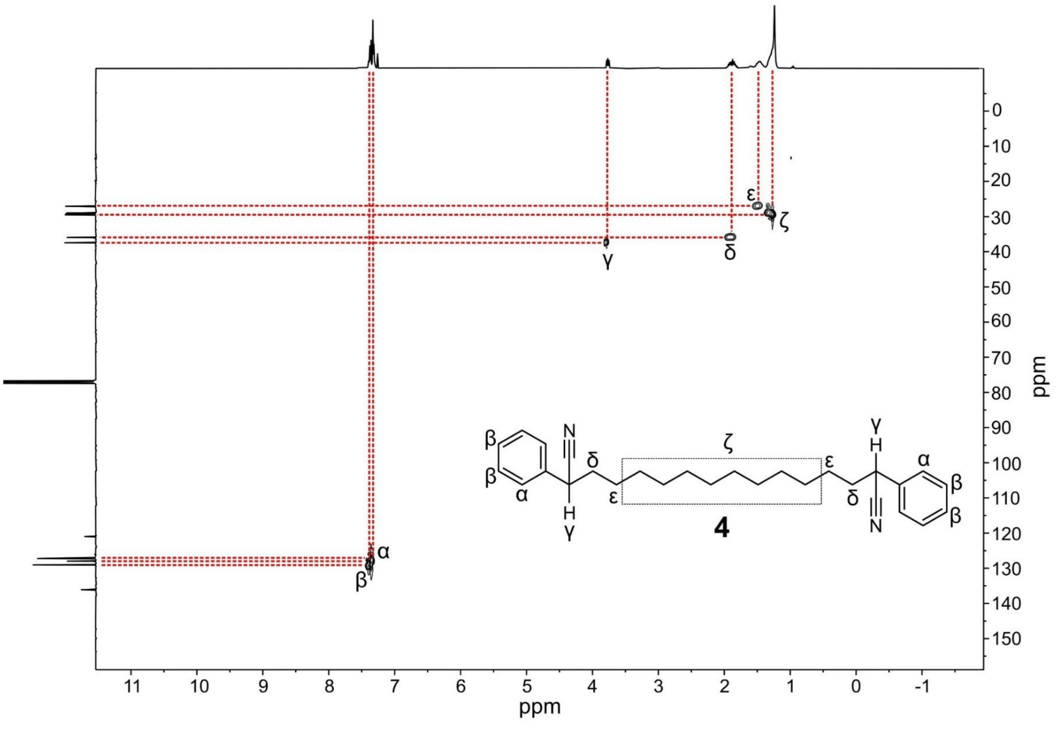

Figure S14. HSQC spectrum of **4** in $CDCl_3$ (25 °C).

HR-MS (ESI-TOF) analysis of 4

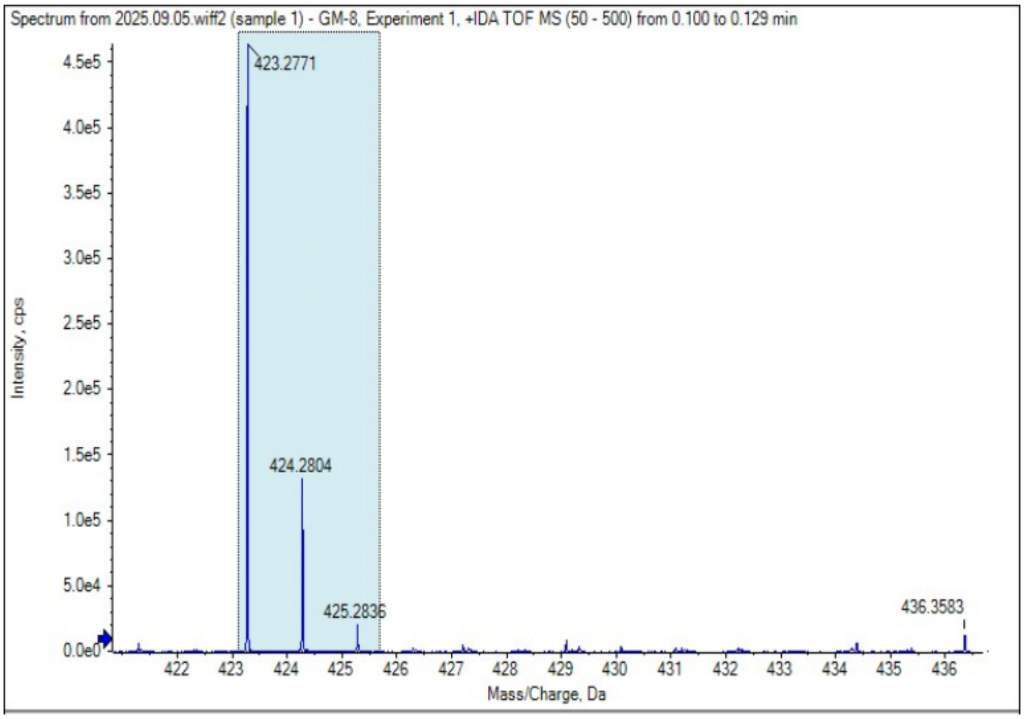

Figure S15. HR-MS (ESI-TOF) of **4**, (positive mode).

DOSY spectrum of 5 mM 4

In a 1.5 mL vial, **1**H_2 (1.34 mg, 0.0028 mmol) was dissolved in 550 μL of a 5 mM solution of **3** (0.0028 mmol) in $CDCl_3$. The vial was heated to 50 °C overnight to assure complete decarboxylation, and then a ^{1}H-DOSY of the solution was recorded.

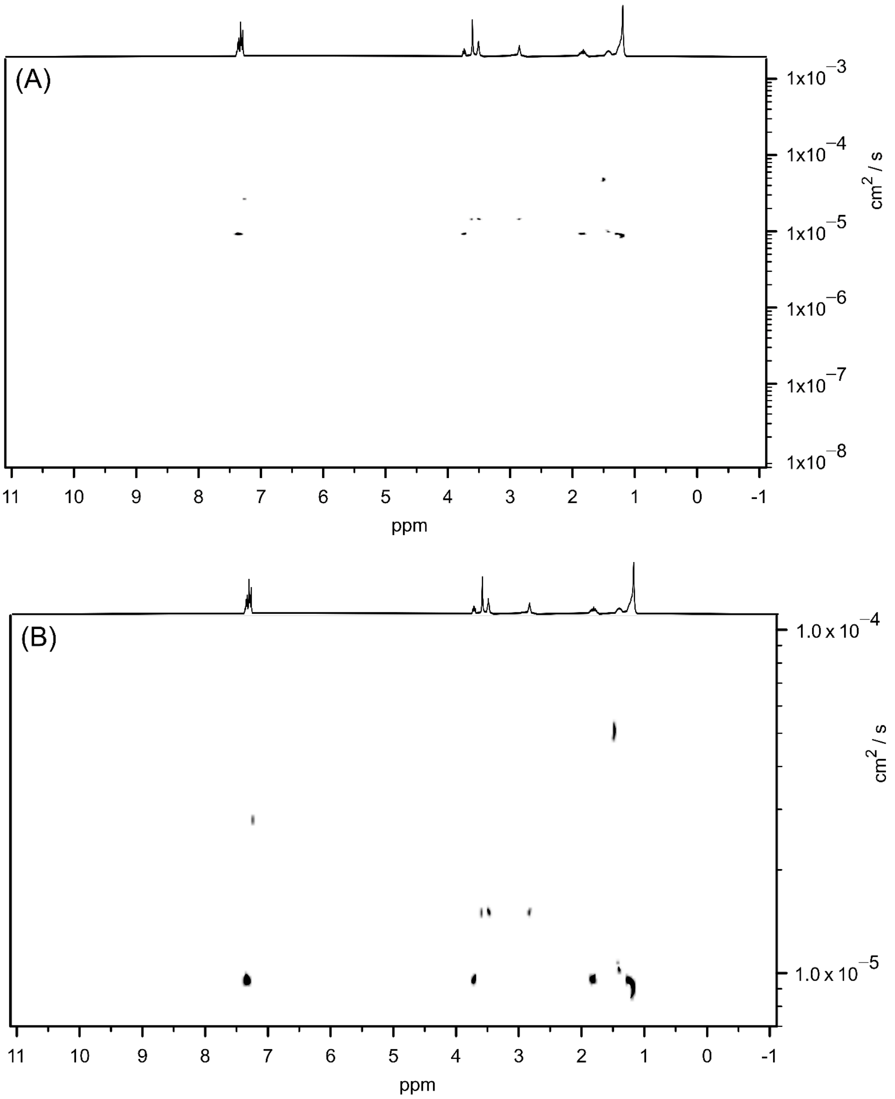

Figure S16. ^{1}H-DOSY spectrum of **4**, obtained by the acid-base reaction between **1**H_2 and **3**, and subsequent decarboxylation of the divalent ACA. The spectrum was recorded in $CDCl_3$ (25 °C). An enlargement of the region of observed D values of the ^{1}H-DOSY spectrum plotted in *A*, is given in *B*.

At 5 mM concentration, the diffusion coefficient recorded for **4** was 8.91 10^{-6} cm^2/s. The other signals in the spectrum are related to diamine **3** (1.46·10^{-5} cm^2/s), $CDCl_3$ and water.

DOSY spectrum of 5 mM 3

In a NMR tube, 300 µL of 10 mM **3** in $CDCl_3$ was diluted with 300 µL of $CDCl_3$. A ^{1}H-DOSY of the resulting 5 mM solution of **3** was then recorded. At 5 mM concentration, the diffusion coefficient recorded for **3** was $1.56\cdot10^{-5}$ cm^2/s.

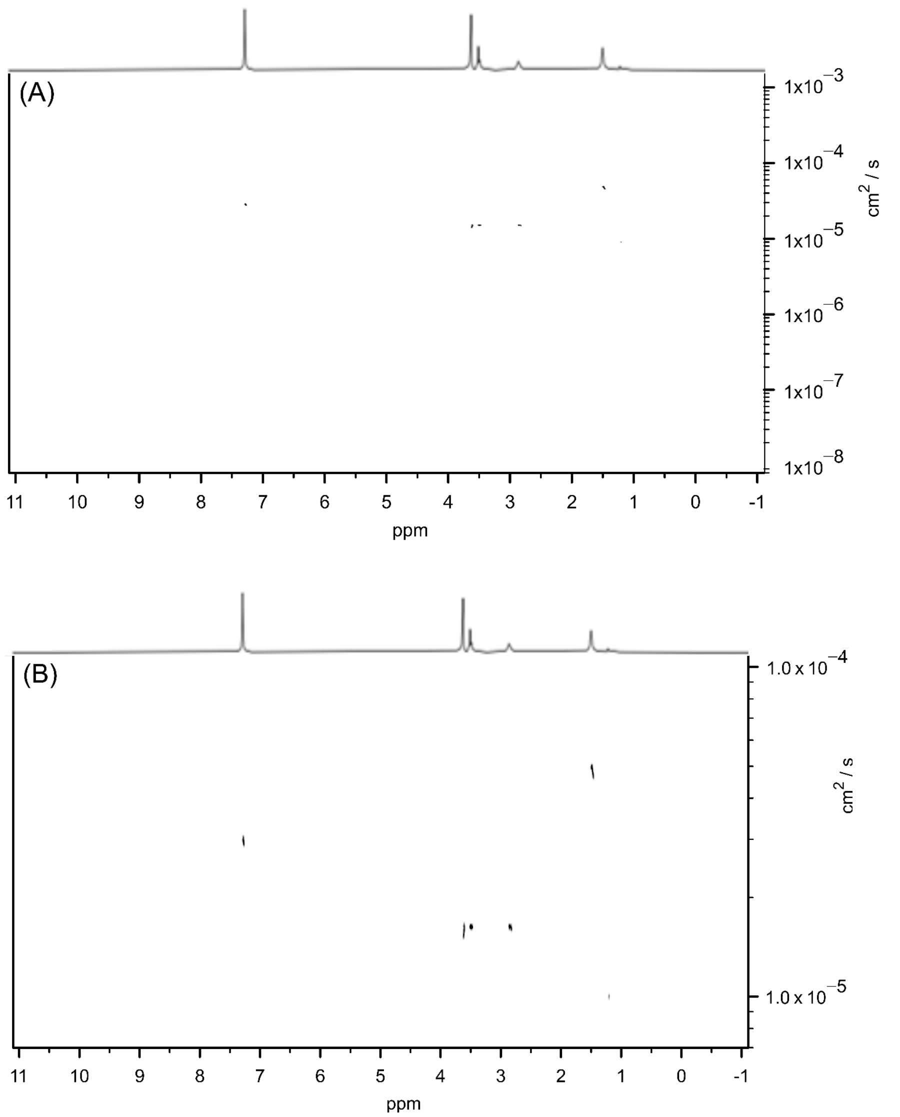

Figure S17. ^{1}H-DOSY spectrum of **3**, recorded in $CDCl_3$ (25 °C). An enlargement of the region of observed *D* values of the ^{1}H-DOSY spectrum plotted in *A*, is given in *B*.

^{1}H-NMR monitoring of a 1:2 mixture of $1H_2$ (10 mM) and 5 (20 mM)

In a 1.5 mL vial, **1**H_2 (2.69 mg, 0.0055 mmol) was dissolved in 550 µL of 20 mM **5** (0.011 mmol) in $CDCl_3$. The composition of the solution was monitored by ^{1}H-NMR (Figure S18). Due to the observed slowness of the processes involved, 120 µL of CD_3CN were added, and ^{1}H NMR monitoring was again performed (see Figure S19).

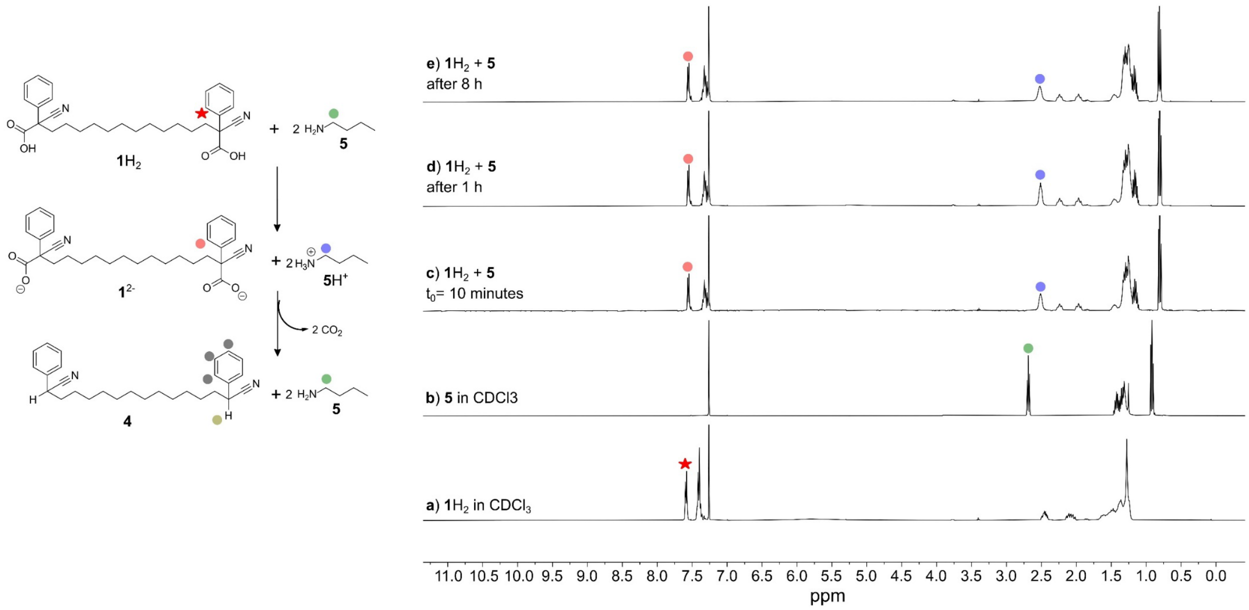

Figure S18. ^{1}H-NMR spectra, in $CDCl_3$ (25 °C), of 10 mM **1**H_2 (trace *a*), 20 mM **5** (trace *b*), and of a 1:2 mixture of **1**H_2 (10 mM) + **5** (20 mM), after 10 min, 1h, and 8 h from mixing (traces *c* – *e*, respectively).

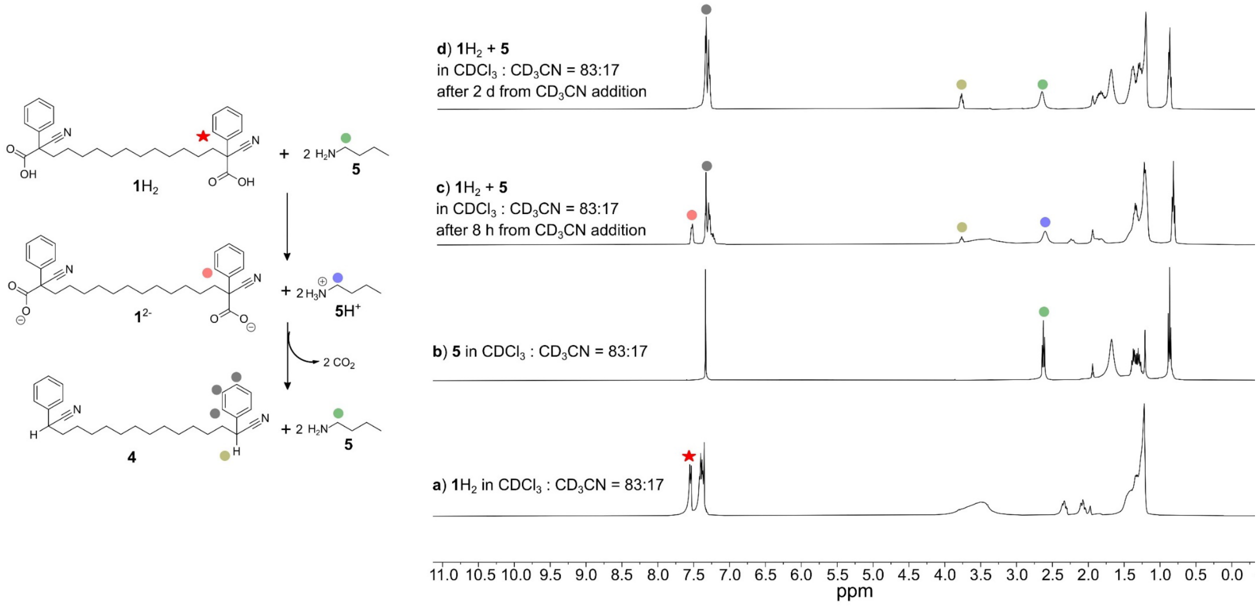

Figure S19. ^{1}H-NMR spectra in $CDCl_3/CD_3CN$ 83:17 (v/v) at 25 °C, of 10 mM **1**H_2 (*a*), 20 mM **5** (*b*), and of a 1:2 mixture of **1**H_2 (10 mM) and **5** (20 mM), after 8 h (*c*), and 2 d (*d*), from CD_3CN addition.

^{1}H-NMR monitoring of a 1:1 mixture of **1**H_2 (10 mM) and 3 (10 mM)

In a 1.5 mL vial, **1**H_2 (2.69 mg, 0.0055 mmol) was dissolved in 550 μL of 10 mM **3** (0.0055 mmol), in $CDCl_3$. For ^{1}H-NMR monitoring of this system see Figure 2 in the main text.

DOSY spectrum of 1H_2 (200 mM) + 3 (200 mM)

In a 1.5 mL vial, **1** (53.7 mg, 0.11 mmol) was dissolved in 550 μL of $CDCl_3$. Then, **3** (16.1 μL, 0.11 mmol) was added to the solution, and a DOSY spectrum of the system was recorded right after. In this assembly, a diffusion coefficient of $5.66 \cdot 10^{-7}$ cm^2/s was recorded for **1**H_2.

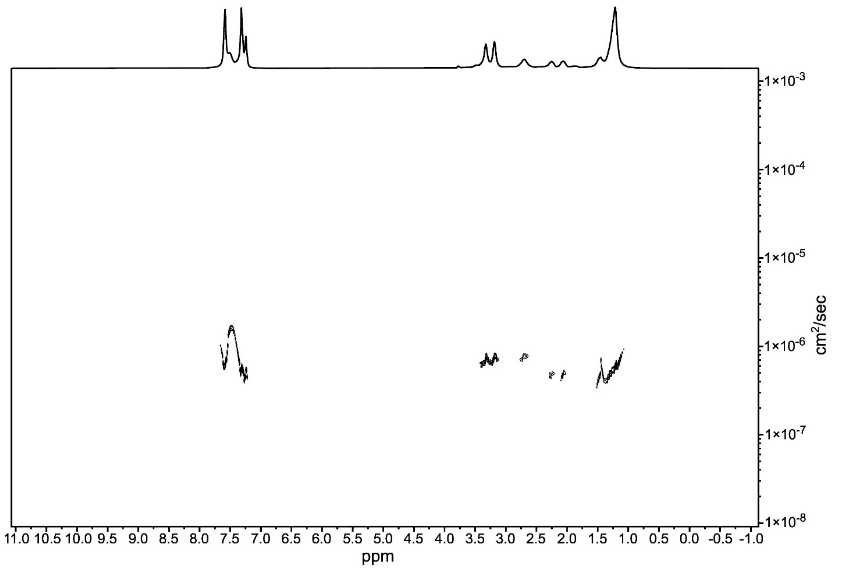

Figure S20. DOSY spectrum of the system **1**H_2 + **3**, both at 200 mM, in $CDCl_3$ (25 °C).

DOSY spectrum of 1H2 (150 mM) + 3 (150 mM)

In a 1.5 mL vial, **1**H_2 (40.3 mg, 0.083 mmol) was dissolved in 550 µL of $CDCl_3$. Then, **3** (12.0 µL, 0.083 mmol) was added to the solution, and a DOSY spectrum of the system was recorded right after. In this assembly, a diffusion coefficient of $7.28 \cdot 10^{-7}$ cm^2/s was recorded for **1**H_2.

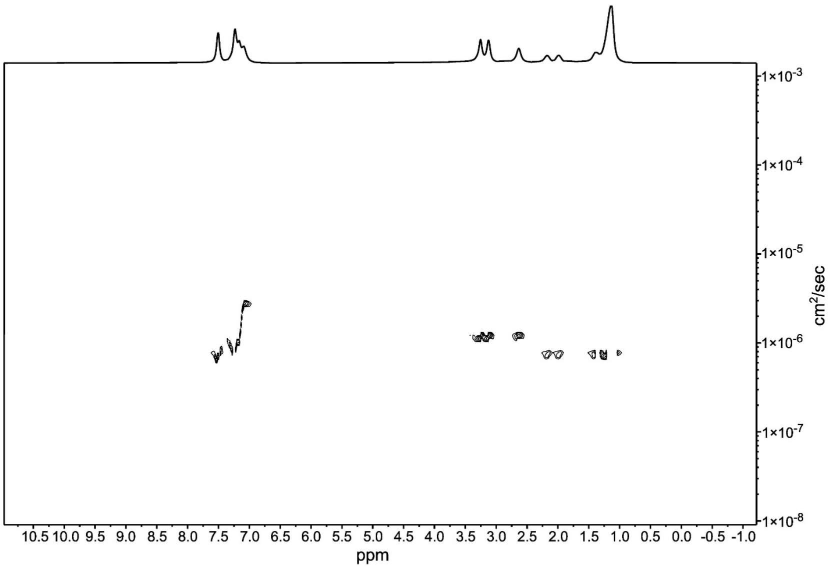

Figure S21. DOSY spectrum of the system **1**H_2 + **3**, both 150 mM, in $CDCl_3$ (25 °C).

DOSY spectrum of 1H2 (100 mM) + 3 (100 mM)

In a 1.5 mL vial, **1**H_2 (26.9 mg, 0.055 mmol) was dissolved in 550 µL of $CDCl_3$. Then **3** (8.0 µL, 0.055 mmol) was added to the solution, and a DOSY spectrum of the system was recorded right after. In this assembly, a diffusion coefficient of $1.20 \cdot 10^{-6}$ cm^2/s was recorded for **1**H_2.

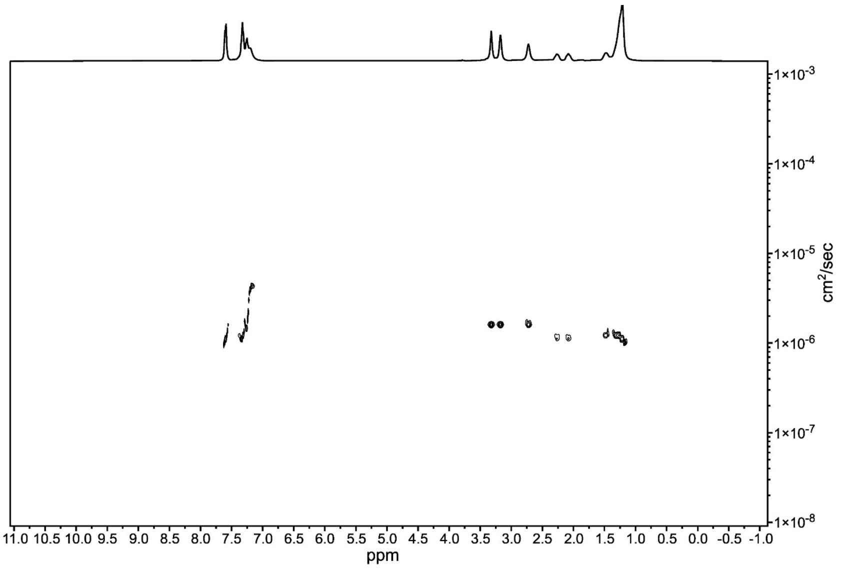

Figure S22. DOSY spectrum of the system **1**H_2 + **3**, both 100 mM, in $CDCl_3$ (25 °C).

DOSY spectrum of 1H_2 (80 mM) + 3 (80 mM)

In a 1.5 mL vial, **1**H_2 (21.5 mg, 0.044 mmol) was dissolved in 550 μL of $CDCl_3$. Then, **3** (6.4 μL, 0.044 mmol) was added to the solution, and a DOSY spectrum of the system was recorded right after. In this assembly, a diffusion coefficient of $1.69\cdot10^{-6}$ cm^2/s was recorded for **1**H_2.

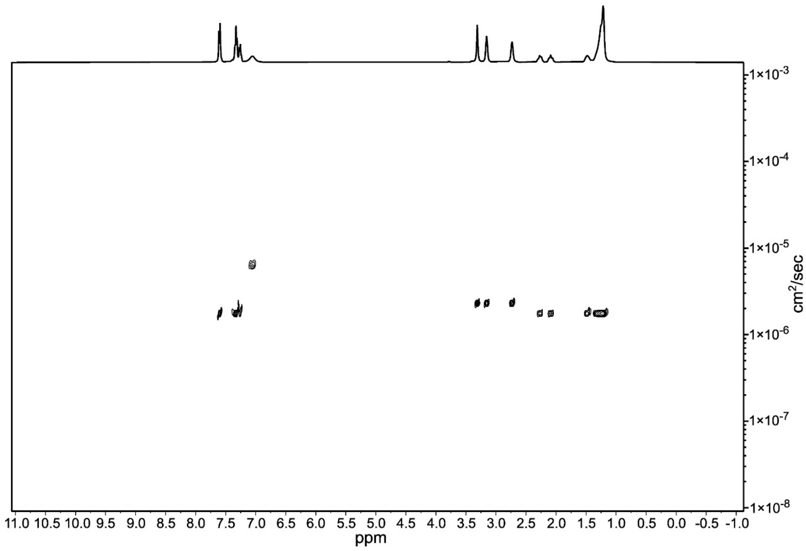

Figure S23. DOSY spectrum of the system **1**H_2 + **3**, both 80 mM, in $CDCl_3$ (25 °C).

DOSY spectrum of 1H_2 (60 mM) + 3 (60 mM)

In a 1.5 mL vial, **1**H_2 (16.1 mg, 0.033 mmol) was dissolved in 550 μL of $CDCl_3$. Then, **3** (4.8 μL, 0.033 mmol) was added to the solution, and a DOSY spectrum of the system was recorded right after. In this assembly, a diffusion coefficient of $2.28\cdot10^{-6}$ cm^2/s was recorded for **1**H_2.

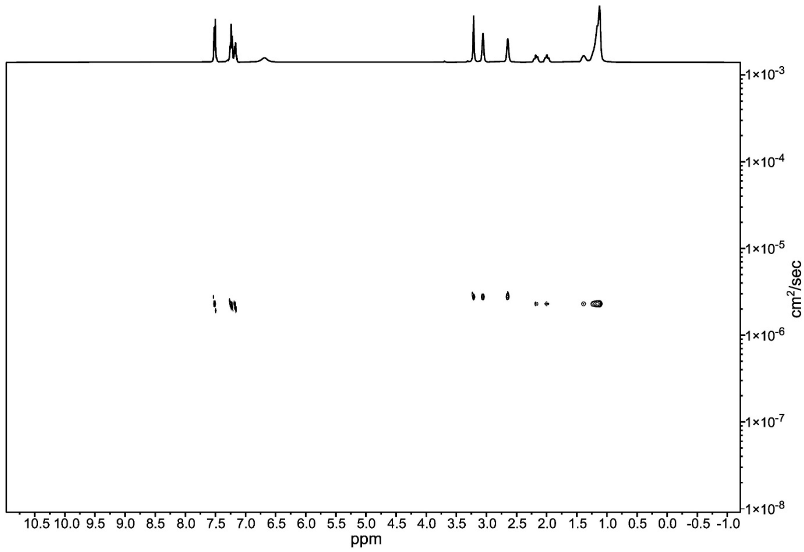

Figure S24. DOSY spectrum of the system **1**H_2 + **3**, both 60 mM, in $CDCl_3$ (25 °C).

DOSY spectrum of $1H_2$ (50 mM) + 3 (50 mM)

In a 1.5 mL vial, **1**H_2 (13.4 mg, 0.028 mmol) was dissolved in 550 μL of $CDCl_3$. Then **3** (4.0 μL 0.028 mmol) was added to the solution, and a DOSY spectrum of the system was recorded right after. In this assembly, a diffusion coefficient of $2.34 \cdot 10^{-6}$ cm^2/s was recorded for **1**H_2.

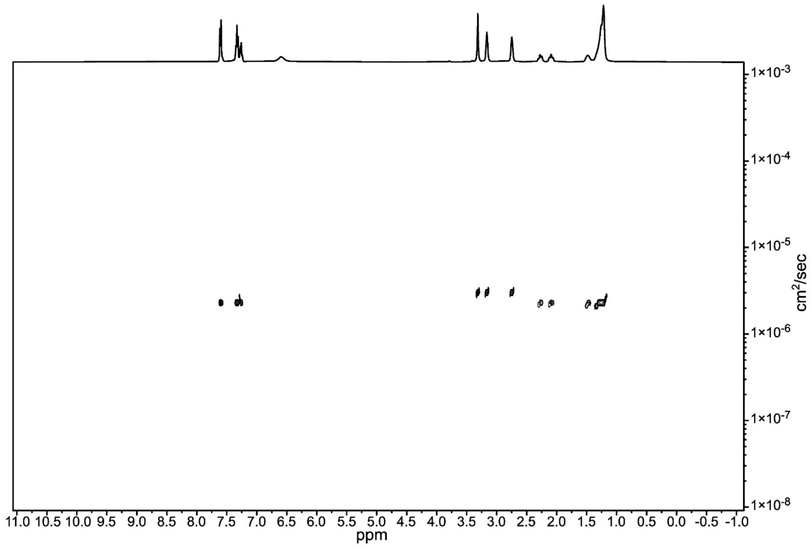

Figure S25. DOSY spectrum of the system **1**H_2 + **3**, both 50 mM, in $CDCl_3$ (25 °C).

DOSY spectrum of $1H_2$ (30 mM) + 3 (30 mM)

In a 1.5 mL vial, **1**H_2 (8.1 mg, 0.017 mmol) was dissolved in 550 μL of $CDCl_3$. Then, **3** (2.5 μL, 0.017 mmol) was added to the solution, and a DOSY spectrum of the system was recorded right after. In this assembly, a diffusion coefficient of $5.67 \cdot 10^{-6}$ cm^2/s was recorded for **1**H_2.

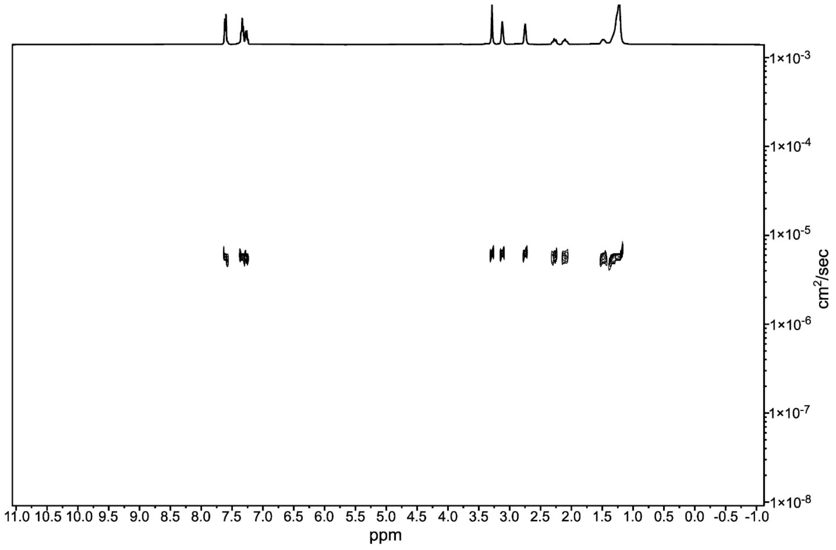

Figure S26. DOSY spectrum of the system **1**H_2 + **3**, both 30 mM, in $CDCl_3$ (25 °C).

DOSY spectrum of $1H_2$ (10 mM) + 3 (10 mM)

In a 1.5 mL vial, **1**H_2 (2.69 mg, 0.0055 mmol) was dissolved in 550 µL of 10 mM of **3** (0.0055 mmol) in $CDCl_3$. A DOSY spectrum of the system was recorded right after, and, in this assembly, a diffusion coefficient of $5.78 \cdot 10^{-6}$ cm^2/s was recorded for **1**H_2.

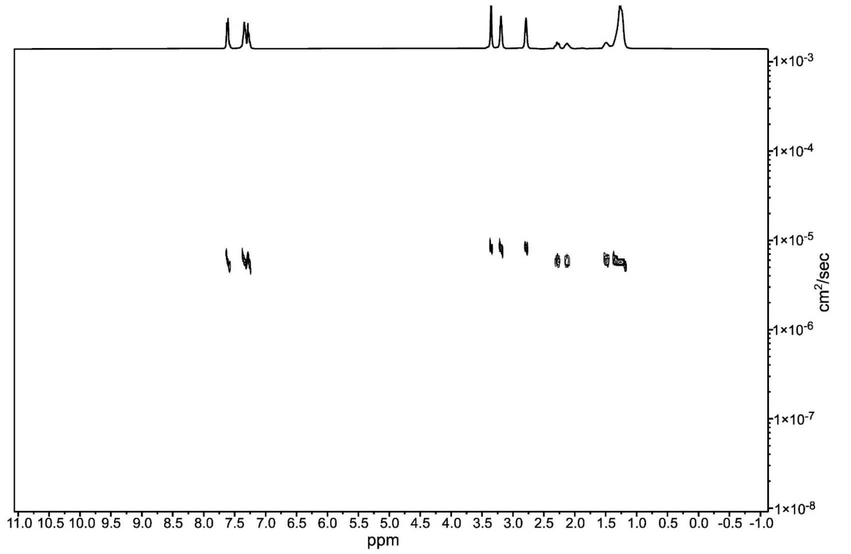

Figure S27. DOSY spectrum of the system **1**H_2 + **3**, both 10 mM, in $CDCl_3$ (25 °C).

DOSY spectrum of $1H_2$ (5 mM) + 3 (5 mM)

In a 1.5 mL vial, **1**H_2 (1.34 mg, 0.00275 mmol) was dissolved in 550 µL of 5 mM **3** (0.00275 mmol), in $CDCl_3$. A DOSY spectrum of the system was recorded right after, obtaining in this assembly a diffusion coefficient of $5.77 \cdot 10^{-6}$ cm^2/s for **1**H_2.

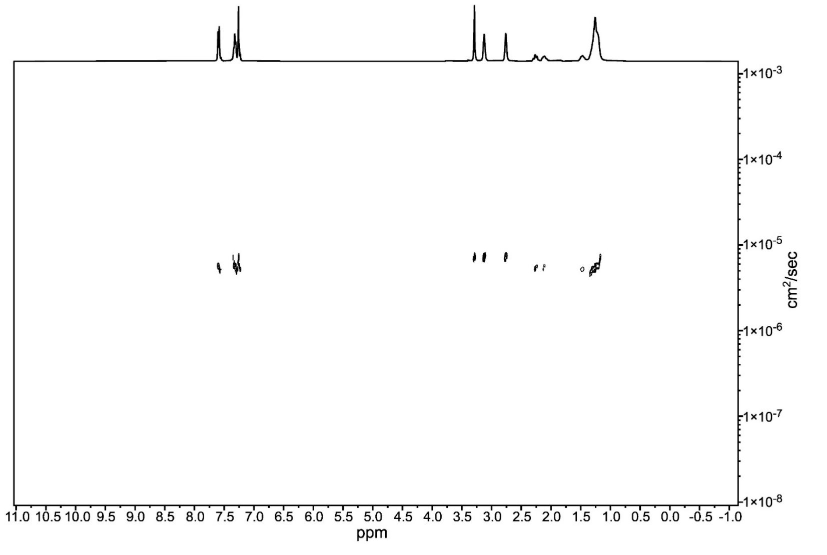

Figure S28. DOSY spectrum of the system **1**H_2 + **3**, both 5 mM, in $CDCl_3$ (25 °C).

^{1}H-NMR monitoring of a 1:1 mixture of 1H$_2$ (100 mM) and 3 (100 mM)

In a 1.5 mL vial **1**H$_2$ (26.9 mg, 0.055 mmol) was dissolved in 550 µL of 100 mM **3** (0.055 mmol), in CDCl$_3$. The composition of the solution was monitored at RT by ^{1}H-NMR.

Decarboxylation was completed after 5 days, however diamine **3** was not completely recovered. Signal integration, in fact, revealed a residue of 36% of the total amount of **3** in solution. The rest of the diamine was involved in the heterophase formed 2 days after **1**H$_2$ addition.

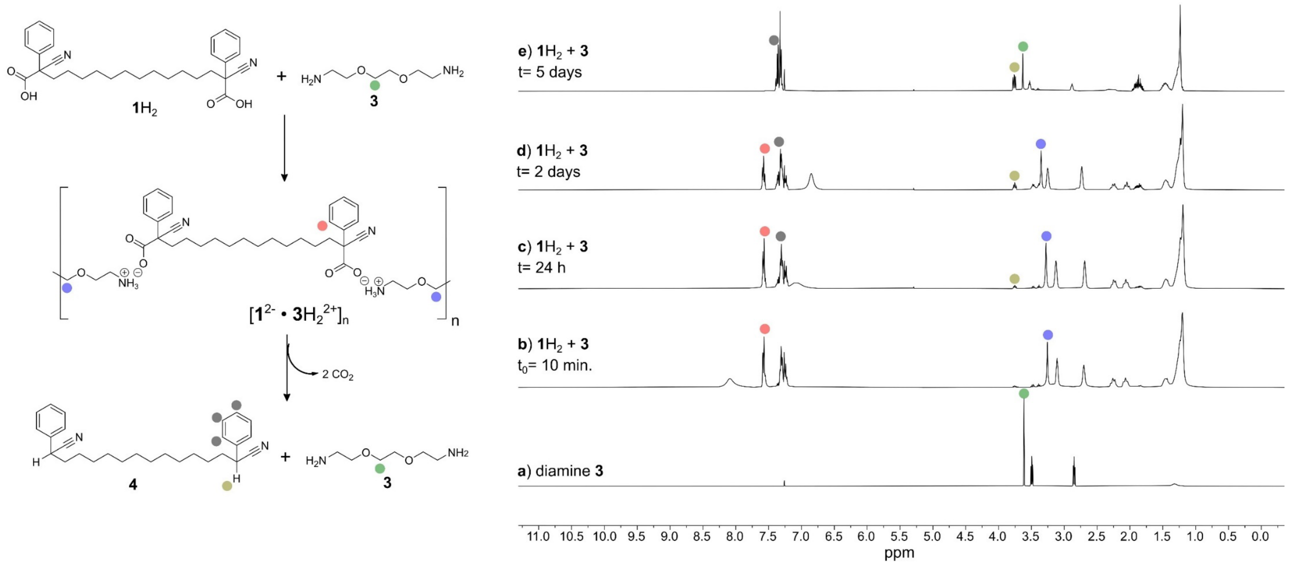

Figure S29. ^{1}H-NMR spectra, of diamine **3** alone (trace *a*), and 10 min (trace *b*), 24 h (trace *c*), 2 h (trace *d*), and 5 d (trace *e*) after mixing with **1**H$_2$, in CDCl$_3$ (25 °C).

^{1}H-NMR monitoring of a 1:1 mixture of 1H$_2$ (100 mM) and 3 (100 mM) at 50 °C

In a 1.5 mL vial, **1**H$_2$ (26.9 mg, 0.055 mmol) was dissolved in 550 µL of CDCl$_3$. Then, **3** (8.0 µL, 0.055 mmol) was added to the solution. ^{1}H-NMR monitoring for 24 hours at 25 °C was carried out (Figure S30). To avoid the formation of any precipitate, the solution was subsequently heated at 50 °C for 24 hours, then a ^{1}H-NMR spectrum was recorded.

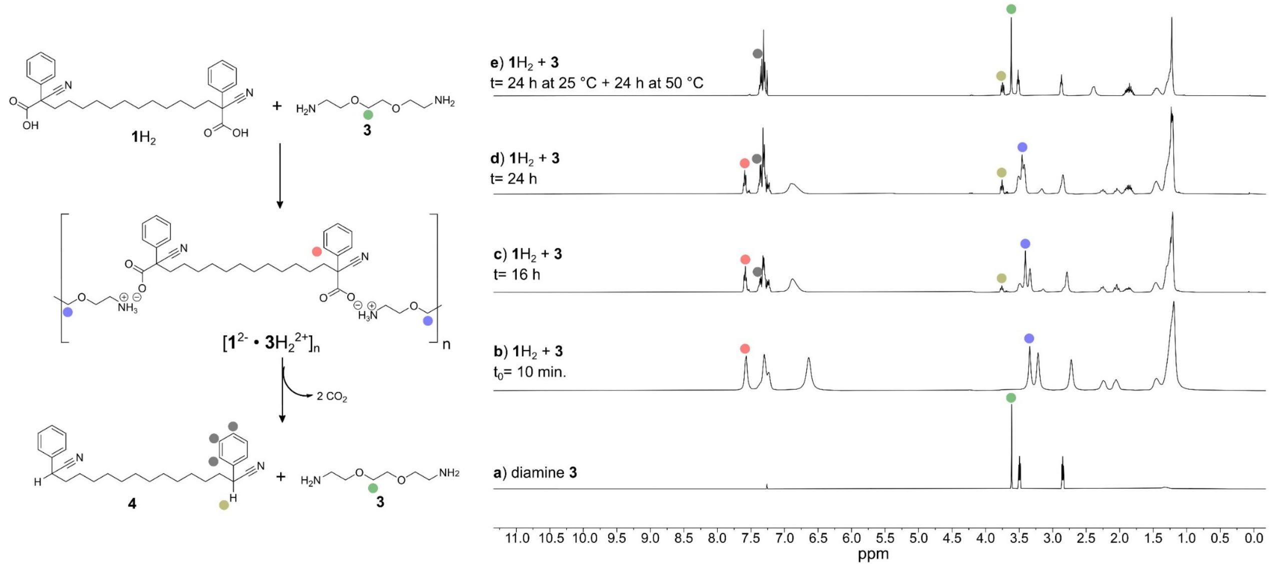

Figure S30. ^{1}H-NMR spectra in CDCl$_3$ of diamine **3** alone (trace *a*), and in the presence of equimolar **1**H$_2$ after 10 min, 16 h, and 24 h, respectively, from mixing (traces *b–d*), at 25 °C. The ^{1}H-NMR spectrum taken after an additional day at 50 °C is reported in trace *e*.

DOSY monitoring of a 1:1 mixture of 1H$_2$ (100 mM) and 3 (100 mM) at 50 °C

In a 1.5 mL vial, **1**H$_2$ (26.9 mg, 0.055 mmol) was dissolved in 550 µL of CDCl$_3$. Then, **3** (8.0 µL, 0.055 mmol) was added to the solution. DOSY spectra were recorded after 20 min, and after 24 h at 25 °C, and finally after one additional day at 50 °C.

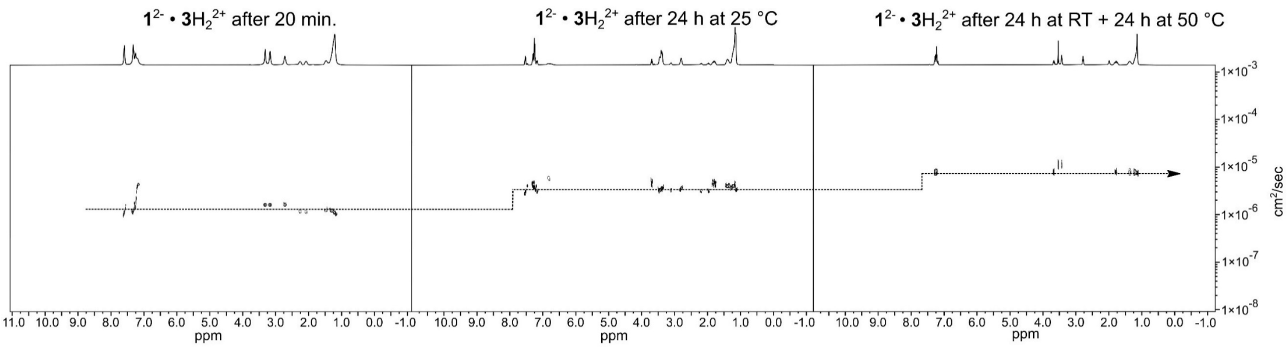

Figure S31. From left to right, DOSY spectra of 100 mM **1**H$_2$ with 100 mM **3**, recorded after 20 min and 24 h at 25 °C, and additional 24 h at 50 °C, in CDCl$_3$,. The spectra were recorded at RT. The stepped arrow shows how the diffusion coefficient of **1**H$_2$ increases over time.

The diffusion coefficient of **1**H_2 increased after 24 hours at RT from $1.20\cdot10^{-6}$ cm^2/s to 3.22 10^{-6} cm^2/s, indicating a progressive disassembling of the ionic supramolecular polymer. After 24 hours of heating, the diffusion coefficient reached a plateau value of $7.66\cdot10^{-6}$ cm^2/s. At this point, divalent ACA **1**H_2 was totally replaced by the decarboxylated product **4**, and the ionic supramolecular assembly was no longer present in solution. It should be pointed out that this recorded diffusion coefficient was higher than the one obtained for **1**H_2 alone at 100 mM (see page S10), of $4.55\cdot10^{-6}$ cm^2/s, suggesting that the diacid was present in a heavier form (a situation different from the one at 5 mM, in which **4** and **1**H_2 had very similar values). Thus, divalent ACA **1**H_2 at high concentrations exists in the form of short oligomers, such as dimers, trimers, and tetramers, held together by hydrogen bonds involving terminal carboxylic moieties.

^{1}H-NMR monitoring of a 1:1 mixture of 1H_2 (200 mM) and 3 (200 mM) at 50 °C

In a 1.5 mL vial, **1**H_2 (53.7 mg, 0.11 mmol) was dissolved in 550 µL of $CDCl_3$. Then, **3** (16.0 µL, 0.11 mmol) was added to the solution. ^{1}H-NMR monitoring for 24 hours at 25 °C was carried out (Figure S32). To avoid the formation of any precipitate, the solution was subsequently heated at 50 °C for 24 hours, then a ^{1}H-NMR spectrum was recorded.

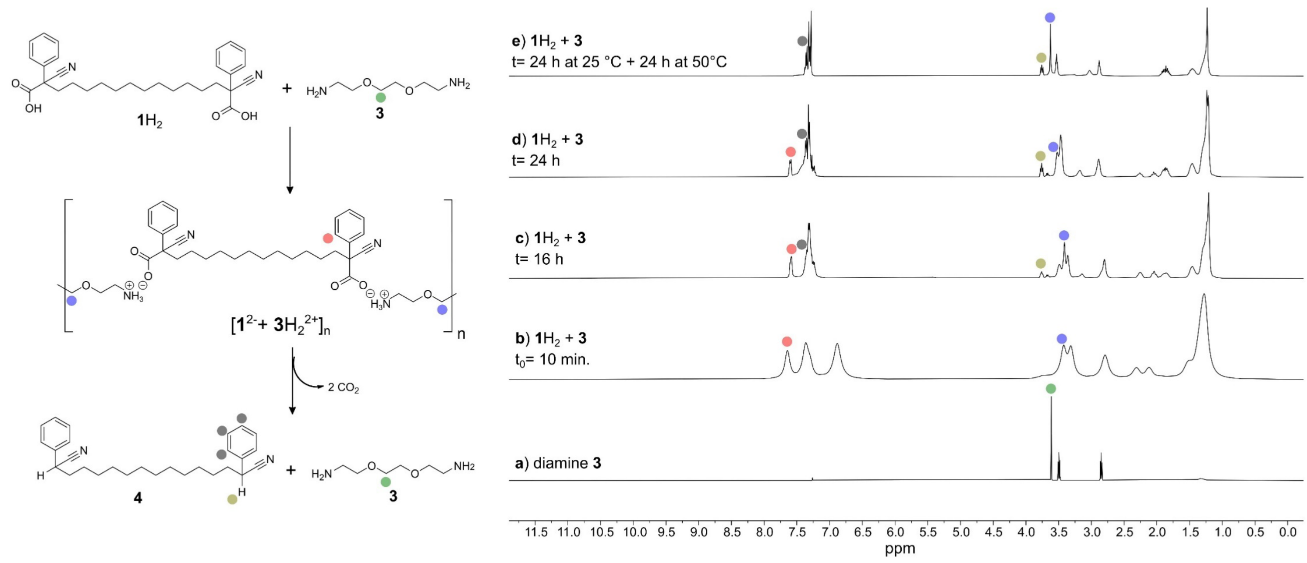

Figure S32. ^{1}H-NMR spectra in $CDCl_3$ of diamine **3** alone (trace *a*), and in the presence of equimolar **1**H_2 after 10 min, 16 h, and 24 h, respectively, from mixing (traces *b–d*), at 25 °C. The ^{1}H-NMR spectrum taken after an additional day at 50 °C is reported in trace *e*.

^{1}H-NMR titration of 5 with 2H ($CDCl_3$, RT)

The association constant K between ammonium **5**H^+ and carboxylate **2**$^-$ (see Scheme S1) has been evaluated by ^{1}H-NMR titration of 550 µL of 2.0 mM butylamine (**5**) with 21.5 mM **2**H in $CDCl_3$, at room temperature. The titrant solution was also 2.0 mM in **5**, in order to avoid dilution of the latter in the titration. In fact, under these conditions, decarboxylation of **2**H is so slow that it can be considered negligible. Acid-base reactions of **2**H with **5** turned out to be immediate.

In Figure S33, the ^{1}H NMR stacked plots of the titration of 2.0 mM **5** with **2**H are reported.

The corresponding titration curves obtained by monitoring of the chemical shifts of the α-methylene and methyl groups of **5** are reported in Figure S34 (top and bottom curves, respectively).

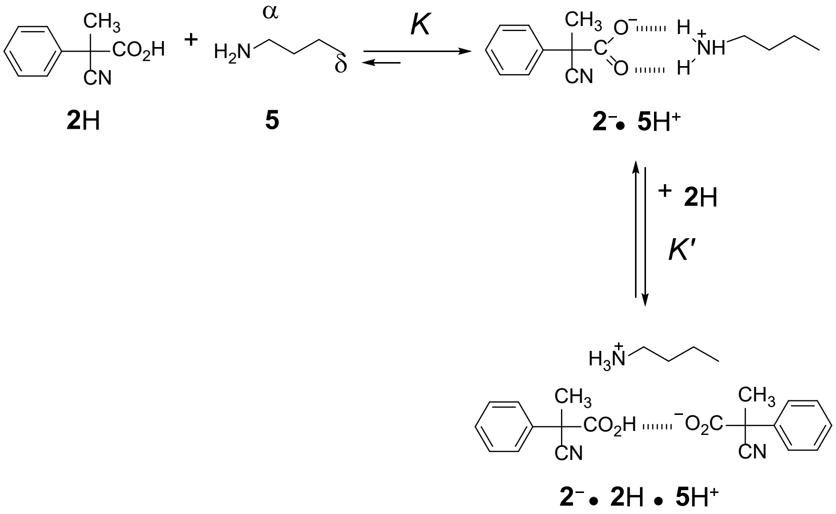

Scheme S1. Equilibria involved in the titration of **5** with **2**H.

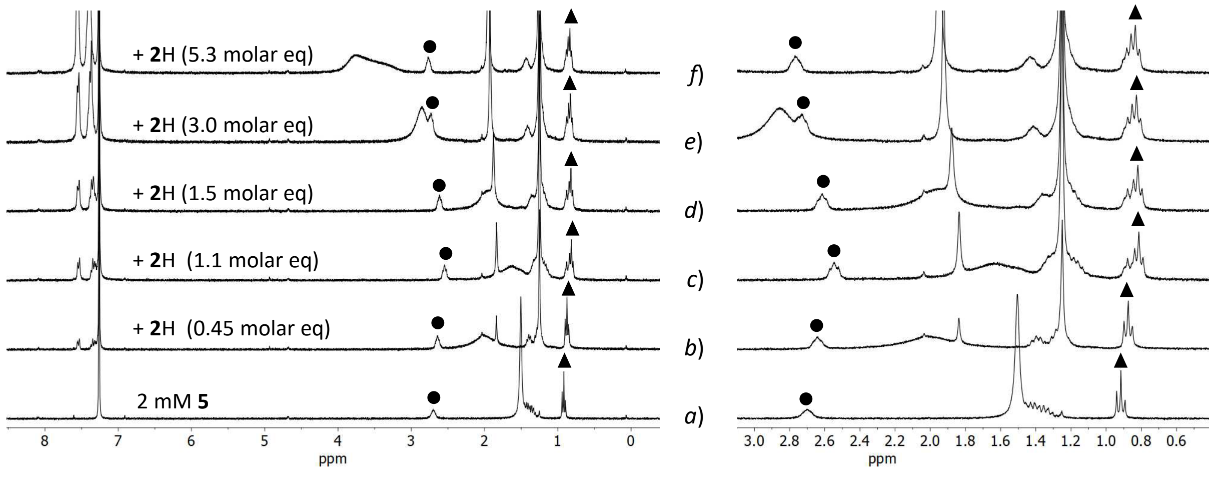

Figure S33. ^{1}H-NMR titration of 2.0 mM **5** with **2**H in $CDCl_3$, (25 °C). ^{1}H-NMR spectra (enlargements of the aliphatic region are shown on the right), registered in the absence of added **2**H (trace *a*), and at increasing concentration of **2**H (traces *b-f*), as reported. The signals of α–methylene and of methyl protons of **5** are marked with circles and triangles, respectively.

In the first phase of the titration a 1:1 strong binding is observed, with a linear profile until a cusp appears exactly at 1 molar equivalent of **2**H added. This is compatible with a very high *K* value ≥ 10^5 M^{-1} for the first equilibrium (note that the concentration of **5** is hold constant in solution and equal to 2.0 mM). When **2**H is added in excess, a nice 1:1 binding isotherm is observed, which is very likely ascribable to the vertical equilibrium depicted in Scheme S1, where the excess acid solvates the ion pair. This behavior was already reported for a similar system.[S1] In the present case, a satisfactorily accurate value of $K' = 1.3\times10^3$ M^{-1} can be estimated for such equilibrium.

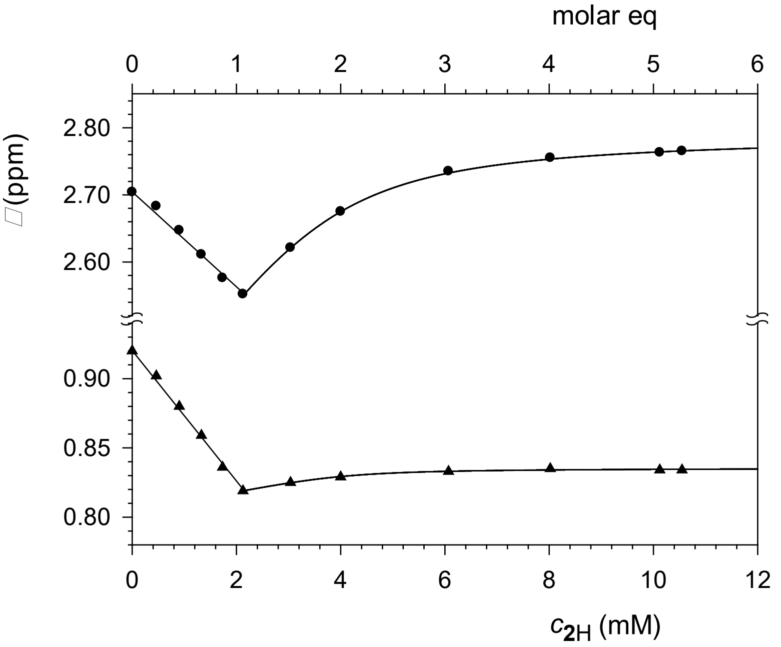

Figure S34. ^{1}H-NMR titration of 2.0 mM **5** with **2**H in $CDCl_3$, (25 °C). Observed δ values of the α–methylene protons (circles; top), and of the methyl protons of **5** (triangles; bottom) *vs* total concentration of added **2**H (c_{2H}). The points are experimental and the 1:1 binding isotherms are calculated from best-fit values of the parameters (see text below).

Results obtained from nonlinear least-square fitting of experimental data (observed δ values of the α–methylene protons of **5** (circles; top) in the presence of an excess of **2**H) to the binding isotherm for the formation of the ternary complex $\mathbf{2}^-\cdot\mathbf{2}H\cdot\mathbf{5}H^+$ from 1:1 binding of **2**H to the ion pair $\mathbf{2}^-\cdot\mathbf{5}H^+$:

		StdErr	CV(%)	dependencies
$\delta_{\text{ternary complex}}$ (ppm)	2.790	2×10^{-3}	0.1	0.8484
K' (M^{-1})	1.3×10^3	69	5	0.8484

Results obtained from nonlinear least-square fitting of experimental data (observed δ values of the methyl protons of **5** (triangles; bottom) in the presence of an excess of **2**H) to the binding isotherm for the formation of $\mathbf{2}^-\cdot\mathbf{2}H\cdot\mathbf{5}H^+$ from 1:1 binding of **2**H to the ion pair $\mathbf{2}^-\cdot\mathbf{5}H^+$:

		StdErr	CV(%)	dependencies
$\delta_{\text{ternary complex}}$ (ppm)	0.835	6×10^{-4}	0.1	0.7585
K' (M^{-1})	2.8×10^3	940	33	0.7585

^{1}H-NMR titration of 3 with 2H ($CDCl_3$, RT)

The strength of the binding between **3**H$^+$ and carboxylate **2**$^-$ (see Scheme S2) has been estimated by ^{1}H-NMR titration of 550 μL of 2.0 mM diamine **3** with 41.0 mM **2**H in $CDCl_3$, at room temperature. The titration was carried as out as rapidly as possible to avoid decarboxylation of **2**H. The experiment lasted 1 hour during which no significant decarboxylation was detected in the ^{1}H-NMR spectra. The titrant solution was also 2.0 mM in **3**, in order to avoid dilution of the latter in the titration. Acid-base reactions of **2**H with **3** turned out to be immediate.

In Figure S35, the ^{1}H NMR stacked plots of the titration of 2.0 mM **3** with **2**H are reported.

The corresponding titration curves obtained by monitoring the chemical shifts of the *α*-methylene and *δ*-methylene groups of **3** are reported in Figure S36 (bottom and top curves, respectively).

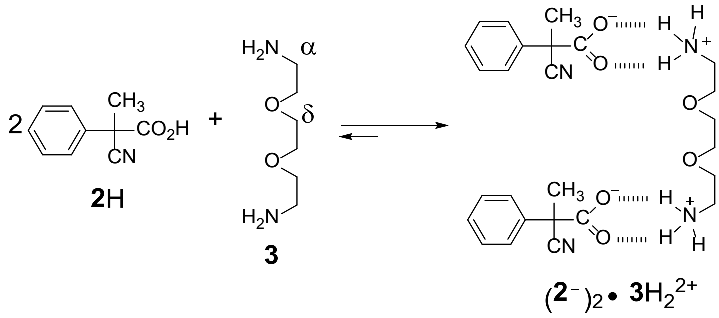

Scheme S2. Equilibria involved in the titration of **3** with **2**H up to 2 molar quivalents of monofunctional acid **2**H added to difunctional amine **3**.

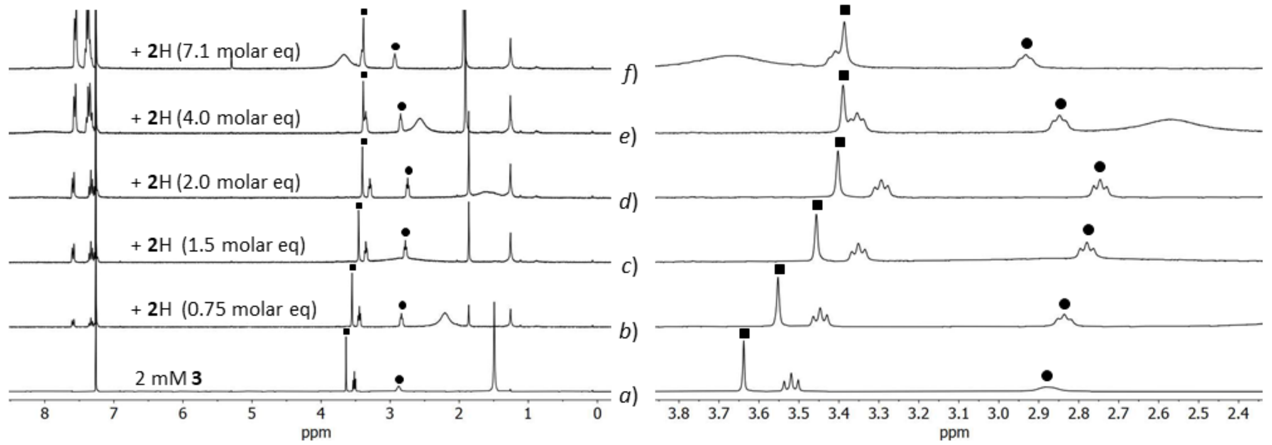

Figure S35. ^{1}H-NMR titration of 2.0 mM **3** with **2**H in $CDCl_3$, (25 °C). ^{1}H-NMR spectra (enlargements of the aliphatic region are shown on the right), registered in the absence of added **2**H (trace *a*), and at increasing concentration of **2**H (traces *b-f*), as reported. The signals of *α*–methylene and *δ*–methylene of **3** are marked with squares and triangles, respectively.

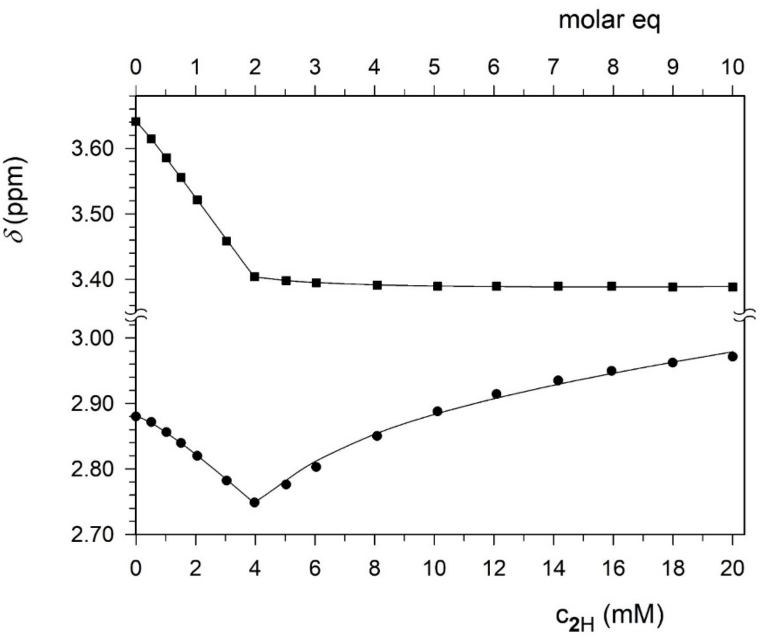

Figure S36. ^{1}H-NMR titration of 2.0 mM **3** with **2**H in $CDCl_3$, (25 °C). Observed values of the δ−methylene protons (squares; top), and of the α−methylene protons of **3** (circles; bottom) *vs* total concentration of added **2**H. The lines are merely guides to the eye.

The two profiles in Figure S36 show a cusp at exactly 2 molar equivalents of **2**H added to **3**. It is a clear evidence that the 1:2 binding between **3**H_2^{2+} and **2**$^{-}$ is strong and the related association constants are too high to measure by ^{1}H NMR.

Theory of ring-chain equilibria for an equimolar mixture of A-A + B-B monomers

Let us consider an equimolar mixture of two monomers A-A and B-B at the initial concentrations $[M_1^{AA}]_0 = [M_1^{BB}]_0 = c_0$. The two functional groups, A and B, can react with each other in a reversible addition reaction, whose equilibrium constant, *K*, is independent of the length of the chains to which they are attached.

Initially, we only consider the process of linear polymerization. Three types of linear oligomers can form: M_i^{AA}, constituted by *i* molecules of A-A and (i -1) molecules of B-B, and having A groups at both ends; M_i^{BB}, constituted by *i* molecules of B-B and (i -1) molecules of A-A, and having B groups at both ends; and M_i^{AB} constituted by an equal number, *i*, of molecules of A-A and B-B and having an A group at one end and a B group at the other end. Accordingly, the mass balance equation in terms of monomeric units is given by eq S1

$$2c_0 = \left[M_1^{AA}\right]_0 + \left[M_1^{BB}\right]_0 = \sum_{i=1}^{\infty}(2i-1)\left(\left[M_i^{AA}\right] + \left[M_i^{BB}\right]\right) + (2i)\left[M_i^{AB}\right] \tag{S1}$$

Since $[M_1^{AA}]_0 = [M_1^{BB}]_0$, the polymer system contains equal amounts of the two odd oligomers with the same *i* value, i.e. $[M_i^{AA}] = [M_i^{BB}]$.

The overall association equilibrium between A and B groups is shown in eq S2, where *x* is the *extent of reaction*.

$$\underset{2c_0(1-x)}{\text{A}} \quad + \quad \underset{2c_0(1-x)}{\text{B}} \quad \overset{K}{\rightleftharpoons} \quad \underset{2c_0x}{\text{AB}} \tag{S2}$$

From eqs S1 and S2, and considering the expression for the equilibrium constant *K*, eq S3 is easily obtained.

$$\sum_{i=1}^{\infty}(2i-1)\left(\left[\mathrm{M}_i^{\mathrm{AA}}\right]+\left[\mathrm{M}_i^{\mathrm{BB}}\right]\right)+(2i)\left[\mathrm{M}_i^{\mathrm{AB}}\right]=\frac{x}{K(1-x)^2} \tag{S3}$$

It is well known that the term $x/(1-x)^2$, appearing in the right-hand of eq S3, is the sum of the series shown in eq S4 for $0 \leq x < 1$ (it could be easily proved by expanding the function in Mclaurin series).

$$\frac{x}{(1-x)^2}=\sum_{i=1}^{\infty} i x^i \tag{S4}$$

Substituting eq S4 into eq S3, and splitting the series into the sum of odd and even terms, we obtain eq S5.

$$\sum_{i=1}^{\infty}(2i-1)\left(\left[\mathrm{M}_i^{\mathrm{AA}}\right]+\left[\mathrm{M}_i^{\mathrm{BB}}\right]\right)+(2i)\left[\mathrm{M}_i^{\mathrm{AB}}\right]=\frac{1}{K}\sum_{i=1}^{\infty}(2i-1)\,x^{2i-1}+(2i)x^{2i} \tag{S5}$$

Eq S5 clearly shows that the distribution of odd and even linear oligomers is related to the extent of reaction by eqs S6 and S7, respectively.

$$\left[\mathrm{M}_i^{\mathrm{AA}}\right]=\left[\mathrm{M}_i^{\mathrm{BB}}\right]=\frac{x^{2i-1}}{2K} \tag{S6}$$

$$\left[\mathrm{M}_i^{\mathrm{AB}}\right]=\frac{x^{2i}}{K} \tag{S7}$$

In contrast to odd oligomers, even oligomers can undergo reversible cyclization by association of their own chain ends, according to eq S8, where C_i is the cyclic oligomer constituted by an equal

number, i, of molecules of A-A and B-B, and $K_{(intra)i}$ is the corresponding intramolecular equilibrium constant.

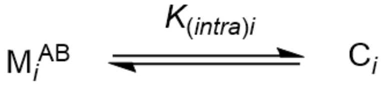

(S8)

By allowing cyclization of even linear oligomers the mass balance equation becomes as follows:

$$2c_0 = \sum_{i=1}^{\infty}(2i)[\mathrm{C}_i] + \sum_{i=1}^{\infty}(2i-1)\left([\mathrm{M}_i^{\mathrm{AA}}] + [\mathrm{M}_i^{\mathrm{BB}}]\right) + (2i)[\mathrm{M}_i^{\mathrm{AB}}] \quad \text{(S9)}$$

Note that eqs S3-S7 are still valid with the caveat that x now represents *the extent of reaction in the chain fraction* only.

Substituting eq S7 into the expression for the equilibrium constant $K_{(intra)i}$, and introducing the well-known concept of equilibrium effective molarity, EM_i, defined by eq S10, eq S11 is easily obtained.

$$EM_i = \frac{K_{(intra)i}}{K} \quad \text{(S10)}$$

$$[\mathrm{C}_i] = EM_i x^{2i} \quad \text{(S11)}$$

For a series of strainless cyclic oligomers formed from long chains obeying Gaussian statistics (say longer than 25-30 skeletal bonds), Jacobson and Stockmayer (JS) showed that the equilibrium effective molarity, EM_i, is given by eq S12 (eq 2 in the main text), where the factor B corresponds to the effective molarity of C_1 (see refs 22, 23 of the main text).

$$EM_i = Bi^{-5/2} \tag{S12}$$

Substitution of eq S12 into eq S11 gives the distribution for a series of strainless cyclic oligomers (eq S13).

$$[\mathrm{C}_i] = Bi^{-5/2}x^{2i} \tag{S13}$$

Substituting eqs S3 and S13 into eq S9, and dividing both sides by 2, the mass balance equation in terms of monomeric units of one kind is obtained (eq S14, eq 3 in the main text).

$$c_0 = B\sum_{i=1}^{\infty} i^{-3/2}x^{2i} + \frac{x}{2K(1-x)^2} \tag{S14}$$

Solving eq S14 for x allows the calculation of the distribution of linear and cyclic oligomers at equilibrium by eqs S6, S7, and S13. The two terms in the right-hand side of eq S14 represent the amount of monomer of one kind in the ring (c_r) and chain (c_c) fractions, respectively.

In the case of a very high association constant, eq S14 predicts the phenomenon of the critical concentration (c_{crit}). It is the monomer concentration below which all the monomers go into the cyclic fraction, and above which the cyclic fraction stops growing and all the monomers go into the chain fraction. This phenomenon occurs because a very large K value assures that c_c is always negligible until x is very close to 1, whereas c_r converges to $c_{crit} = 2.612B$ for x tending to 1, meaning that the cyclic fraction, in contrast with the chain fraction, can only contain a limited number of monomeric units.

For a given mixture of oligomers, N_i, there are two types of degree of polymerization: the number-average degree of polymerization, DP_n, defined as the weighted mean of the degrees of polymerization of the oligomers weighted by their mole fractions (or their number of molecules) (eq

S15), and the weight-average degree of polymerization, *DP*, defined as the weighted mean of the degrees of polymerization of the oligomers weighted by their weight fractions (or their yield) (eq S16).

$$DP_n = \frac{\sum_{i=1}^{\infty} i[N_i]}{\sum_{i=1}^{\infty}[N_i]} \tag{S15}$$

$$DP = \frac{\sum_{i=1}^{\infty} i^2[N_i]}{\sum_{i=1}^{\infty} i[N_i]} \tag{S16}$$

In the case of ring-chain equilibria, although the proportion of rings at high c_0 values may be scarcely significant on a weight basis, they may make a substantial contribution to the total *number* of molecules and, hence, markedly lower the overall number average degree of polymerization. Therefore, the high values of *DP* determined via ^{1}H DOSY necessarily refer to the weight-average degree of polymerization.

Taking into account eq S16, the weight-average degree of polymerization of the ring fraction, DP_r, is given by eq S17.

$$DP_r = \frac{\sum_{i=1}^{\infty}(2i)^2[\mathrm{C}_i]}{\sum_{i=1}^{\infty}(2i)[\mathrm{C}_i]} \tag{S17}$$

Substituting eq S13 into eq S17, eq S18 is obtained (eq 5 in the main text).

$$DP_r = \frac{2\sum_{i=1}^{\infty} i^{-1/2}x^{2i}}{\sum_{i=1}^{\infty} i^{-3/2}x^{2i}} \tag{S18}$$

The weight-average degree of polymerization of the chain fraction (DP_c) is given by eq S19.

$$DP_c = \frac{\sum_{i=1}^{\infty}(2i-1)^2\left(\left[\mathrm{M}_i^{\mathrm{AA}}\right]+\left[\mathrm{M}_i^{\mathrm{BB}}\right]\right)+(2i)^2\left[\mathrm{M}_i^{\mathrm{AB}}\right]}{\sum_{i=1}^{\infty}(2i-1)\left(\left[\mathrm{M}_i^{\mathrm{AA}}\right]+\left[\mathrm{M}_i^{\mathrm{BB}}\right]\right)+(2i)\left[\mathrm{M}_i^{\mathrm{AB}}\right]} \quad \text{(S19)}$$

Substituting eqs S3, S6, and S7 into eq S19, eq S20 is obtained.

$$DP_c = \frac{\sum_{i=1}^{\infty} i^2 x^i}{x/(1-x)^2} \quad \text{(S20)}$$

The sum of the series appearing in the numerator of eq S20 converges to the function $x(1 + x)/(1 - x)^3$ when $0 \leq x < 1$. Thus, eq S20 can be rewritten as eq S21 (eq 6 in the main text).

$$DP_c = \frac{1+x}{1-x} \quad \text{(S21)}$$

The weight-average degree of polymerization of the system, *DP*, is given by the weighted mean of DP_r and DP_c weighted for the corresponding weight fractions, as shown in eq S22 (eq 4 in the main text).

$$DP = \frac{c_r DP_r + c_c DP_c}{c_0} \quad \text{(S22)}$$

Estimation of *B* factor by Mandolini's method

For a large strainless ring constituted by ν identical rotatable bonds of length *l*, the theory predicts that *B* can be calculated by the following equation:[S2]

$$B = \frac{1}{N_A \sigma}\left(\frac{3}{2\pi C_\infty \nu l^2}\right)^{3/2} \qquad \text{(S23)}$$

where N_A is the Avogadro constant, σ is the symmetry number of the ring, and C_∞ is the characteristic ratio in the limit of high chain length, which measures the stiffness of a chain. Mandolini has shown that taking $C_\infty = 8$ and $l = 1.54\times10^{-9}$ dm (corresponding to the Csp^3-Csp^3 bond length), eq S23 fits a large body of experimental effective molarities of large rings in common organic solvents, even if some of the rotatable bonds are not CC bonds (refs. 24c,d,f of the main text). Introducing these values and the values of the other known parameters, eq S23 becomes eq S24 (eq 7 in the main text)

$$B = 6.63\frac{\nu^{-3/2}}{\sigma} \qquad \text{(S24)}$$

The value of σ is equal to 1 for a C_1 ring of the type *c*-(A-B), and equal to 2 for C_1 rings of the type *c*-(A-A) and *c*-(A-AB-B).

For the cycle C_1 under examination, there are 24 rotatable bonds (see Figure S37) and σ = 2, thus the estimated value of *B* is 2.8×10^{-2} mol L^{-1}.

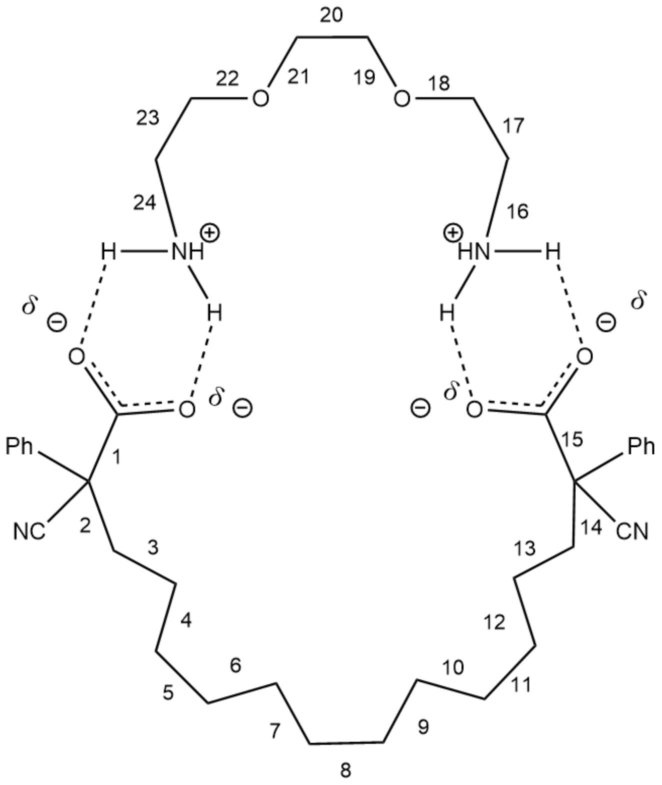

Figure S37. Presumed structure of the C1 ring in the present work. The rotatable single bonds are numbered from 1 to 24.

Optimization of the *B* factor and the intermolecular equilibrium constant *K*

Unfortunately, eq S14 cannot be solved for x, and, thus, it is not possible to express the weight-average degree of polymerization in explicit form, i.e. $DP = f(c_0)$. Accordingly, standard non-linear least squares programs cannot be used to optimize B and K.

We proceeded as follows. Our first goal was to build tables of numerical values of DP as a function of c_0 for fixed values of B and K. To this end, we considered both c_0 and DP as functions of x, as expressed by eqs. S14 and S22, respectively. However, evaluation of c_0 and DP by eqs S14 and S22 requires the computation of the series in eqs S25 and S26 for any given value of x in the range [0,1].

$$\sum_{i=1}^{\infty} i^{-1/2} x^{2i} \tag{S25}$$

$$\sum_{i=1}^{\infty} i^{-3/2} x^{2i} \tag{S26}$$

Series in eqs S25 and S26 are of the type shown in eq S27 with $y = x^2$, where s is a rational number and y is a real number between 0 and 1; they have been studied by Truesdell (ref. 25 in the main text).

$$\phi(y,s) = \sum_{i=1}^{\infty} i^{-s} y^{i} \tag{S27}$$

The series in eq S27 is rapidly convergent for y values near 0 but slowly convergent for y values near 1. Truesdell showed that the series $\phi(y,s)$ as defined in eq S27 can also be calculated by the series in eq S28, where $\Gamma(z)$ and $\zeta(z)$ are the Gamma function and the Riemann zeta function, respectively (both the functions are implemented in the software program for symbolic computation MATLAB).

$$\phi(y,s) = \Gamma(1-s)(-\ln y)^{s-1} + \sum_{i=0}^{\infty} \zeta(s-i) \frac{(\ln y)^{i}}{i!} \tag{S28}$$

The series in eq S28, in contrast to the series in eq S27, is rapidly convergent for y values near 1 but slowly convergent for y values near 0; thus, the two series complement each other for the evaluation of $\phi(y,s)$ in the y range [0, 1]. Table S1 reports the values of the sums of the series in eqs. S25 and S26 for selected values of x (and $x^2 = y$). The entries in Table S1 were computed by means of either eq S27 or eq S28, and are correct to the fourth decimal figure.

By the data in Table S1, tables of numerical values of *DP* as a function of c_0 for fixed values of *B* and *K* can be easily calculated. Values of $DP_{(calc)}$ that exactly correspond to the c_0 values in Table 1 of the main text can be obtained with good accuracy by linear interpolation.

Table S1.

x	x^2	$\sum_{i=1}^{\infty} i^{-1/2}x^{2i}$	$\sum_{i=1}^{\infty} i^{-3/2}x^{2i}$
0.0000	0.0000	0.0000	0.0000
0.2236	0.0500	0.0518	0.0509
0.3162	0.1000	0.1077	0.1037
0.3873	0.1500	0.1682	0.1587
0.4472	0.2000	0.2339	0.2159
0.5000	0.2500	0.3057	0.2757
0.5477	0.3000	0.3848	0.3383
0.5916	0.3500	0.4723	0.4041
0.6325	0.4000	0.5701	0.4734
0.6708	0.4500	0.6804	0.5468
0.7071	0.5000	0.8061	0.6248
0.7416	0.5500	0.9515	0.7083
0.7746	0.6000	1.1223	0.7982
0.8062	0.6500	1.3272	0.8959
0.8367	0.7000	1.5799	1.0031
0.8660	0.7500	1.9030	1.1226
0.8944	0.8000	2.3376	1.2586
0.9220	0.8500	2.9697	1.4179
0.9487	0.9000	4.0220	1.6144
0.9539	0.9100	4.3307	1.6605
0.9592	0.9200	4.6951	1.7098
0.9644	0.9300	5.1342	1.7628
0.9695	0.9400	5.6780	1.8206
0.9747	0.9500	6.3764	1.8842
0.9798	0.9600	7.3207	1.9556
0.9849	0.9700	8.7018	2.0381
0.9899	0.9800	11.0139	2.1380
0.9950	0.9900	16.2218	2.2717
0.9955	0.9910	17.1827	2.2885
0.9960	0.9920	18.3182	2.3064
0.9965	0.9930	19.6888	2.3255
0.9970	0.9940	21.3888	2.3462
0.9975	0.9950	23.5756	2.3687
0.9980	0.9960	26.5374	2.3938
0.9985	0.9970	30.8764	2.4225
0.9990	0.9980	38.1535	2.4567
0.9995	0.9990	54.5757	2.5017
0.9995	0.9991	57.6083	2.5073
0.9996	0.9992	61.1930	2.5133
0.9996	0.9993	65.5205	2.5196
0.9997	0.9994	70.8890	2.5264
0.99975	0.9995	77.7964	2.5338
0.99980	0.9996	87.1536	2.5421
0.99985	0.9997	100.8647	2.5514
0.99990	0.9998	123.8648	2.5625
0.99995	0.9999	175.7806	2.5771
0.999975	0.99995	249.1994	2.5874
0.999995	0.99999	559.0374	2.6012
0.9999975	0.999995	791.2041	2.6045
0.9999995	0.999999	1770.9931	2.6088
0.9999997	0.9999995	2505.1676	2.6099
0.9999999	0.9999999	5603.5307	2.6113
1.0000000	1.0000000	∞	2.6124

The next goal was to find the B and K values that minimize the sum of the squared residuals shown in eq S29, where DP_i are the experimental values from Table 1 and the sum is extended to the number n of experimental points.

$$S = \sum_{i=1}^{n} \left(DP_i - DP_{(calc)i} \right)^2 \tag{S29}$$

At first, starting from the initial parameters B = 2.8×10^{-2} mol L^{-1} and K = 1.0×10^{5} mol^{-1} L (see estimation of B and K above), we modified the parameters by trial and error to give the best fit, by visual inspection, of the calculated curve to the experimental points. Then, we wanted to verify whether the best B and K values (B = 3.0×10^{-2} mol L^{-1} and K = 4.3×10^{5} mol^{-1} L) we found by visual inspection of the fit, correspond to the minimum of the S function. To this end, we built a 3×3 grid of points on the plane of the coordinates B and K, considering all the points formed by the combinations B = (3.0±0.1) ×10^{-2} mol L^{-1} and K = (4.3±0.1)×10^{5} mol^{-1} L. The point B = 3.0×10^{-2} mol L^{-1} and K =4.3×10^{5} mol^{-1} L is at the center of the grid, and all the other points are on the edge of the grid and surround the center point. Then, we calculated the value of S for each point on the grid to verify whether the center point corresponds to the minimum value of S. It turned out that the point on one vertex of the grid B = 3.1×10^{-2} mol L^{-1} and K = 4.4×10^{5} mol^{-1} L corresponds to a lower S value. Therefore, we built a new 3×3 grid centered at this new point and verified that this point actually corresponds to the minimum of the S function. Accordingly, the optimized values of B and K are B = (3.1±0.1) ×10^{-2} mol L^{-1} and K = (4.4±0.1)×10^{5} mol^{-1} L, where the reported errors refer to the spacings of the grid.

Derivation of eq 1

Derivation of eq 1 in the main text strictly follows a recent approach presented in refs. 18a,b in the main text.

The diffusion coefficient for a spherical particle with a hydrodynamic radius R_H is given by the Stokes-Einstein equation (eq S30), where k is the Boltzmann constant, T the absolute temperature, and η the solvent viscosity.

$$D = \frac{kT}{6\pi\eta R_H} \quad \text{(S30)}$$

R_H can be correlated to the weight-average molecular weight of the polymer, M, via the Rouse-Zimm model (eq S31), where α and b are arbitrary parameters.

$$R_H \sim bM^{\alpha} \quad \text{(S31)}$$

Substituting eq S31 into eq S30, eq S32 is obtained, with A being the adjusted proportionality factor.

$$D = AM^{-\alpha} \quad \text{(S32)}$$

Application of eq S32 to the monomer gives eq S33, where M_0 is the molecular weight of the monomer.

$$D_{monomer} = AM_0^{-\alpha} \quad \text{(S33)}$$

Taking the ratio of eq S33 to eq S32, eq S34 is obtained, where DP, equal to M / M_0, is the weight-average degree of polymerization.

$$\frac{D_{monomer}}{D} = DP^{\alpha} \tag{S34}$$

Solving eq S34 for *DP* yields

$$DP = \left(\frac{D_{monomer}}{D}\right)^{1/\alpha} \tag{S35}$$

For polymer solutions in $CDCl_3$, Junkers et al. (ref. 18a) reported the following α values: poly(methyl methacrilate), 0.56; polystyrene, 0.46; poly(ethylene glycol), 0.45. Analogously, Ruzicka, et al. (ref. 18b) reported the following α values in $CDCl_3$: poly(methyl methacrylate), 0.4767; polystyrene, 0.4985; polybutadiene, 0.5041. Thus, a value of α ~ 0.5 seems appropriate for polymer solutions in $CDCl_3$. Accordingly, eq 1 in the main text is immediately obtained from eq S35.

[S1] See SI at page S25 of Berrocal, J. A.; Biagini, C.; Mandolini, L.; Di Stefano, S. Coupling of the Decarboxylation of 2-Cyano-2-phenylpropanoic Acid to Large-Amplitude Motions: A Convenient Fuel for an Acid–Base- Operated Molecular Switch. *Angew. Chem. Int. Ed.* **2016**, *55*, 6997 – 7001.

[S2] (a) Kuhn, W. Über die gestalt fadenförmiger moleküle in lösungen. *Kolloid Z.* **1934**, *68*, 2 – 15. (b) Jacobson, H.; Stockmayer, W. H. Intramolecular Reaction in Polycondensations. I. The Theory of Linear Systems. *J. Chem. Phys.* **1950**, *18*, 1600 – 1606. (c) Flory, P. J.; Semlyen, J. A. Macrocyclization equilibrium constants and the statistical configuration of poly(dimethylsiloxane) chains. *J. Am. Chem. Soc.* **1966**, *88*, 3209 – 3212. (d) Flory, P. J. *Statistical Mechanics of Chain Molecules*. New York, NY: Interscience; **1969**. (e) Flory, P. J.; Suter, U. W.; Mutter, M. Macrocyclization equilibriums. 1. Theory. *J. Am. Chem. Soc.* **1976**, *98*, 5733 – 5739.
